# DRQ-RTDETR: Degradation-Aware Detail Recovery and Query-Guided RT-DETR for Household Gas Facility Detection

**DOI:** 10.3390/s26154800

**Published:** 2026-07-28

**Authors:** Guanjie Wang, Lanxin Chen, Haoyang Bai, Zixiang Yi, Huaiyu Li, Dongxu Zhang

**Affiliations:** 1 Ulster College, Shaanxi University of Science and Technology, Xi’an 710021, China; 202315020118@sust.edu.cn (G.W.); 202315010201@sust.edu.cn (L.C.); 202315020204@sust.edu.cn (H.B.); 202415020222@sust.edu.cn (Z.Y.); 202215020208@sust.edu.cn (H.L.); 2 College of Mechanical and Electrical Engineering, Shaanxi University of Science and Technology, Xi’an 710021, China

**Keywords:** household gas facility detection, RT-DETR, small-object detection, degradation-aware feature fusion, query selection, geometric refinement, detection transformer

## Abstract

Reliable visual identification of household gas facilities is important for safety inspection, yet images acquired in real indoor environments are frequently affected by cluttered textures, shadows, metallic reflections, stains, dust, motion blur, occlusion, and viewpoint variation. In RT-DETR, these conditions can weaken cross-scale structural cues, reduce the ranking of small-object candidates, and amplify localization errors under strict IoU criteria. We hypothesize that coordinated intervention at feature fusion, query allocation, and box regression can alleviate this coupled failure process without enlarging the decoder-query budget. Accordingly, DRQ-RTDETR integrates degradation-aware detail recovery, small-object-guided query selection, and scale-adaptive geometric refinement. Experiments on a real household gas facility dataset containing 21,813 images and 47,169 instances across eight safety-related categories show that DRQ-RTDETR improves mAP from 0.6168 to 0.6576, mAP50 from 0.7909 to 0.8124, mAP75 from 0.6702 to 0.7136, and mAPsmall from 0.4639 to 0.5247 relative to RT-DETR. The larger gains in mAPsmall and mAP75 indicate that the proposed coordination is particularly effective for weak-response compact components and boundary-sensitive localization in degraded household scenes.

## 1. Introduction

Vision-based analysis can support household gas safety inspections by locating safety-related components in on-site images. Unlike long-distance pipeline inspection or conventional industrial defect detection, this task is performed at close range in uncontrolled indoor environments, where pipes, valves, and connection structures may be fine-grained, densely distributed, and visually intertwined. Shadows, metallic reflections, stains, dust, motion blur, occlusion, and viewpoint changes further weaken discriminative visual evidence and reduce detection reliability [1,2].

Existing studies address real-time DETR-based detection [3], vision-based inspection [4,5,6,7,8], multi-scale DETR representation and feature-hierarchy design [9,10,11,12], degradation-aware feature modeling [13,14,15,16,17], small-object feature and query allocation [18,19,20,21,22], and geometry-aware localization [23,24,25,26,27,28,29]. However, in degraded small-object scenes, the role of multi-scale feature hierarchy has not been sufficiently connected with the subsequent query-accessibility and localization-sensitivity problems: once high-resolution structural cues are weakened or diluted during feature fusion, compact-object tokens may be under-ranked before decoder modeling and remain difficult to localize under strict IoU criteria.

These research directions, however, generally treat the relevant stages separately. Despite these advances, existing methods usually address degraded representation, small-object candidate allocation, and bounding-box optimization as separate problems. This separation is restrictive for household gas-facility detection because errors can propagate sequentially: degradation first weakens fine structural evidence, the weakened responses then reduce the ranking of compact-object tokens during encoder query selection, and the surviving predictions remain highly sensitive to small boundary deviations. The methodological novelty of DRQ-RTDETR therefore lies not in combining three independent enhancement operators, but in coordinating feature recovery, query accessibility, and localization supervision according to this explicit feature-query–localization failure process.

Specifically, DDRF differs from image-restoration-oriented approaches by recovering degradation-affected structure directly in the detection feature space without clean-image supervision. SGQ differs from high-resolution detection heads or query-expansion strategies by reallocating the original K = 300 query budget through a degradation-aware compact-object prior. SAGR further complements these two inference-stage components by applying scale-sensitive geometric supervision only to Hungarian-matched positive queries during training, without introducing an additional inference branch. This coordinated design preserves the end-to-end detection pipeline while targeting three successive failure stages observed in degraded household scenes.

To evaluate this hypothesis, DRQ-RTDETR coordinates degradation-aware detail recovery fusion (DDRF), small-object guided query selection (SGQ), and scale-adaptive geometric refinement (SAGR) at the feature-fusion, query-selection, and localization-supervision stages, respectively. The framework is assessed on a private dataset collected during real household inspections through detector comparisons, staged ablations, class- and scale-specific analyses, qualitative evaluation, and complexity analysis.

The main contributions of this paper are summarized as follows:

1. A coupled feature–query–localization failure process is formulated for household gas-facility detection under mixed visual degradation. This formulation describes how degraded structural cues may reduce compact-object candidate ranking under a fixed query budget and increase scale-sensitive localization errors.
2. A degradation-aware detail recovery fusion module, termed DDRF, is proposed to enhance degradation-affected structural cues directly in the detection feature space, without relying on clean-image supervision or an independent image-restoration branch.
3. Small-object guided query selection and scale-adaptive geometric refinement are introduced to intervene at the query-selection and localization-supervision stages, respectively. SGQ reallocates the unchanged K = 300 decoder-query budget toward weak-response compact candidates, whereas SAGR improves scale-sensitive box regression only during training.
4. Experiments on a real household gas-facility dataset containing 21,813 images and 47,169 instances across eight categories validate the proposed design through detector comparisons, staged ablations, degradation-stratified evaluation, class- and scale-specific analyses, qualitative visualization, and complexity assessment.

## 2. Related Work

### 2.1. Vision-Based Inspection and Detection Transformers

Vision-based inspection is widely used in oil and gas, industrial facilities, and engineering safety monitoring. Deep learning methods have been applied to pipeline anomalies, weld defects, photovoltaic modules, and railway tracks [4,5,6,7,8]. Most of these studies emphasize long-distance pipelines, surface defects, corrosion, leakage-related cues, or comparatively large targets. Household gas facility detection instead requires close-range recognition of fine-grained, structurally connected components under clutter, reflection, staining, and occlusion, motivating task-specific data and detection strategies.

Recent fault-diagnosis studies have further emphasized model reliability under domain shift and limited target-domain data. Siddique et al. [30] developed a class-conditional deep domain-adaptation framework for bearing diagnosis across machines, combining time–frequency representations with class-aware distribution alignment to learn discriminative features that remain transferable under changing operating conditions. Zaman et al. [31] proposed an optimized transfer-learning framework for rotary-machine diagnosis, in which the fine-tuning depth is selected according to target-domain feature separability rather than empirical layer freezing.

Although these studies address vibration-based fault classification rather than image-based object localization, they provide an important deployment perspective: performance obtained within a single data distribution does not necessarily guarantee reliability across machines, devices, or operating environments. The present study addresses a different but related problem by improving robustness to mixed visual degradation within household inspection images through coordinated feature fusion, query allocation, and localization supervision. Cross-region and cross-device domain adaptation remain outside the current scope and are identified as priorities for future work. Other recent reliability-oriented studies have also investigated pipeline leak detection and rotating-machinery diagnosis under multi-domain or variable operating conditions, further supporting the need to examine model behavior beyond a single acquisition setting [32,33].

Detection Transformers formulate object detection as end-to-end set prediction, and subsequent variants improve multi-scale modeling, training stability, and inference efficiency [9,10,11]. A central issue in transformer-based detection is not only whether multi-scale features are used, but also how hierarchical features are introduced into the query-based detection pipeline. High-resolution feature levels retain local edges, corners, thin structures, and compact-object boundaries, whereas low-resolution semantic levels provide category-level and contextual information. However, recent work rethinking the multi-scale feature hierarchy in DETR has shown that simply introducing long multi-scale feature sequences into the encoder may be computationally redundant and suboptimal. Instead, F-DETR uses a heterogeneous-scale multi-branch structure to promote interaction between local and global features and to provide more effective object-container initialization for query-based detection [12].

This perspective is particularly relevant to household gas-facility detection. In degraded indoor scenes, small or boundary-sensitive components such as In_needle, Ft_protection, Pipe_clamp, and Valve often occupy limited pixels and depend on weak local contours. Blur, reflection, stains, dust, occlusion, and background clutter can further suppress these high-resolution structural cues or create misleading high-frequency responses. If the hierarchical feature representation is degraded before query selection, the corresponding compact-object tokens may receive low encoder confidence and fail to enter the fixed decoder-query set. Therefore, multi-scale feature enhancement should not be treated as a generic feature-fusion operation only; it should preserve degradation-affected local details, suppress unreliable responses, and improve the accessibility of compact-object candidates under a limited query budget. Motivated by this observation, DRQ-RTDETR enhances cross-scale structural evidence through DDRF and converts the enhanced high-resolution response into a query-selection prior through SGQ, while keeping the decoder-query number unchanged.

### 2.2. Degradation-Aware Feature Enhancement and Small-Object Query Selection

Blur, reflection, dust, oil stains, and cluttered backgrounds can weaken edges, textures, and local structures in household inspection images. Prior work on degradation-aware restoration, gated fusion, and frequency-domain modeling shows that adaptive feature processing can preserve discriminative structural information [13,14,15,16,17]. DDRF differs from image restoration networks because it neither reconstructs a clean image nor requires clean-image supervision; it operates directly in the detection feature space.

Small-object performance also depends on how limited computational and query resources are allocated. FPN, QueryDet, DQ-DETR, Dome-DETR, and Drone-DETR demonstrate that multi-scale representation and targeted query allocation can improve the accessibility of compact objects [18,19,20,21,22]. SGQ follows this principle while retaining the original decoder-query budget.

### 2.3. Localization Losses for Boundary-Sensitive Objects

After a compact-object candidate enters the decoder, performance depends on the quality of bounding-box supervision. GIoU, DIoU, and CIoU address non-overlap, center distance, and aspect-ratio consistency [23,24], while NWD [25], MPDIoU [26], Inner-IoU [27], Focaler-IoU [28], and Wise-IoU [29] introduce scale-aware, geometry-aware, or difficulty-adaptive constraints. These developments motivate localization objectives that are less sensitive to pixel-level displacement for small targets.

SAGR retains the baseline L1 coordinate-regression term and replaces the original GIoU localization term with scale-adaptive focused-region overlap and corner-normalized refinement. The resulting objective is applied only to Hungarian-matched positive queries during training and introduces no inference branch or additional post-processing.

## 3. Materials and Methods

### 3.1. Household Gas Facility Dataset

The dataset was collected during real household gas-safety inspections and covers eight safety-related classes: Water_heater, Flue, Stove, In_needle, Ft_protection, Rubber_hose, Pipe_clamp, and Valve. The images were acquired in uncontrolled indoor scenes and include defocus or motion blur, shadows, metallic reflections, oil stains, dust, occlusion, background clutter, and viewpoint variation. All instances were annotated with horizontal bounding boxes.

The dataset contains 21,813 images and 47,169 instances, comprising 17,450 training images with 37,688 instances and 4363 validation images with 9481 instances. A scene-level split prevents images from the same household or near-duplicate acquisition sequence from appearing in both subsets. The same split and annotation format were used throughout all experiments.

**Figure 1:**
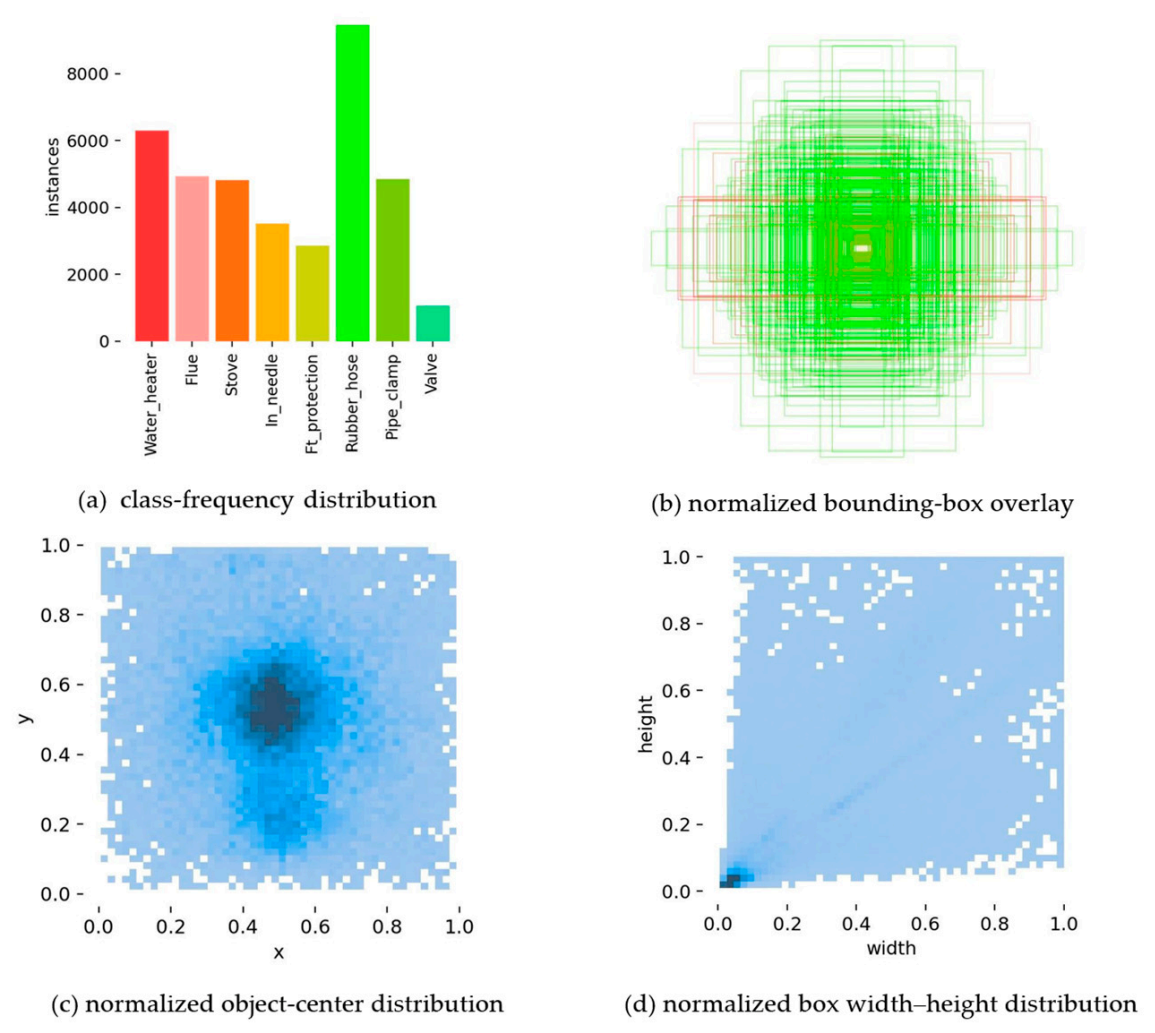
Training-set label statistics and bounding-box distribution of the updated 8-class household gas facility dataset: (**a**) class-frequency distribution; (**b**) normalized bounding-box overlay; (**c**) normalized object-center distribution; and (**d**) normalized box width–height distribution.

Table 1 reports the class-level instance counts, while Figure 1 summarizes the training-set class frequency, object-center distribution, and normalized box dimensions. Rubber_hose is the most frequent class (25.02%), whereas Valve is the least frequent (2.88%).

Figure 1 illustrates the class imbalance and the spatial and scale distributions of the training annotations; the complete training and validation counts are listed in Table 1.

**Figure 2:**
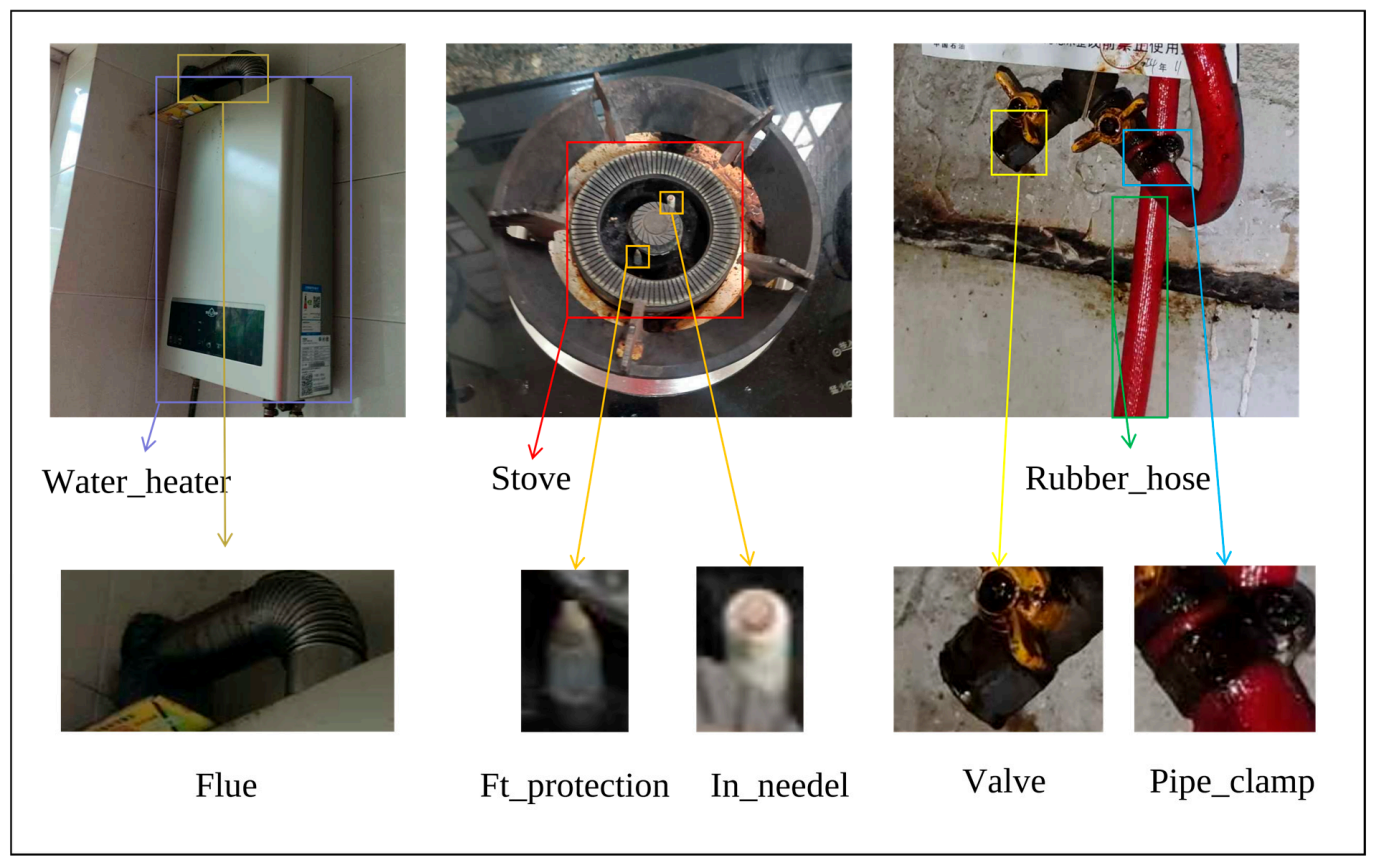
Privacy-preserving examples of real household gas-facility inspection scenes and close-up component regions used to illustrate the dataset acquisition environment. Note: Any non-English characters visible in the original scene are incidental background text captured during real household inspection and are not used for class annotation or detection.

Representative privacy-preserving inspection images and close-up component regions are shown in Figure 2. These examples document the close-range, oblique-view, and cluttered indoor acquisition conditions covered by the dataset. The images were acquired during routine household gas-facility inspections using handheld mobile devices; however, camera metadata, including the device model and camera-to-target distance, were not uniformly available for all samples.

For robustness analysis, the fixed validation set was tagged at the image level for six non-mutually exclusive degradation conditions: blur, reflection or overexposure, stain or dust, occlusion, low illumination or shadow, and background clutter.

### 3.2. Baseline RT-DETR and Problem Formulation

RT-DETR is used as the baseline because its hybrid encoder, encoder-token query selection, and matched-query regression provide the three intervention points required by the proposed hypothesis [3]. DDRF modifies cross-scale fusion, SGQ re-ranks candidates under the original query budget, and SAGR retains the baseline L1 coordinate-regression term and replaces the original GIoU localization term with scale-adaptive focused-region overlap and corner-normalized refinement during training.

In degraded household scenes, weakened local evidence can reduce encoder confidence for compact components. Under a fixed query budget, the corresponding tokens may then be omitted, leaving fewer matched queries for accurate localization. DRQ-RTDETR addresses this coupled feature-query-localization sequence at the three stages where the failures arise.

### 3.3. Overall Architecture of DRQ-RTDETR

**Figure 3:**
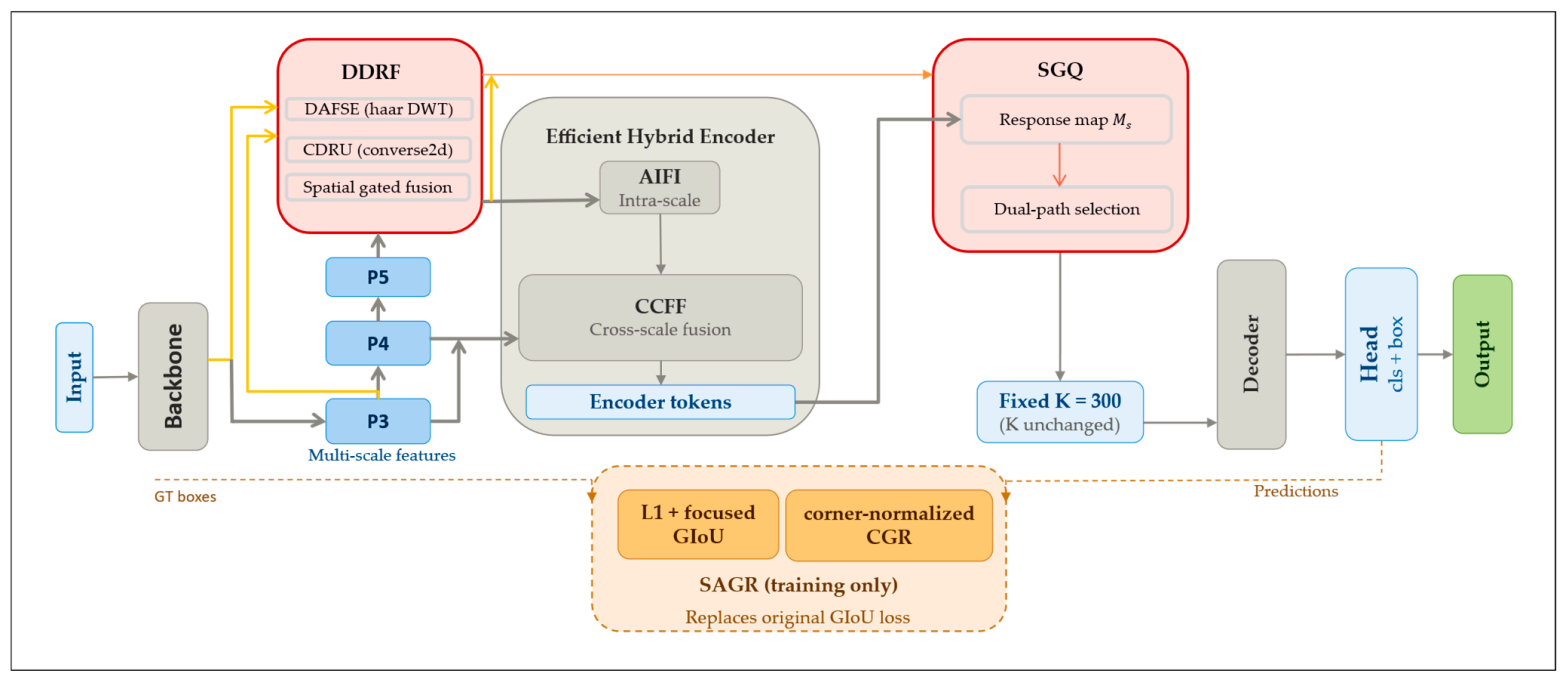
Overall architecture of DRQ-RTDETR and the integration positions of DDRF, SGQ, and SAGR. Solid arrows indicate the forward detection flow, dashed arrows indicate training-only supervision, and colored blocks distinguish the main functional components.

As shown in Figure 3, DRQ-RTDETR preserves the end-to-end RT-DETR pipeline and introduces three task-oriented components at feature fusion, query selection, and box regression.

DDRF recovers detail-aware semantic information and reinforces frequency-structural cues before candidate selection, improving the representation of edges, corners, textures, and local connection structures in degraded images.

SGQ converts the enhanced high-resolution features into a spatial prior and re-ranks encoder candidates through dual-path selection. This increases the likelihood that compact weak-response candidates are retained for decoder processing without increasing the fixed query number.

SAGR keeps the baseline L1 coordinate-regression term and replaces the original GIoU term with scale-adaptive focused-region overlap and corner-normalized refinement for Hungarian-matched positive queries during training.

Together, the three components form a progressive feature-query-localization pipeline while preserving the lightweight, end-to-end structure of the baseline detector.

### 3.4. Degradation-Aware Detail Recovery Fusion

#### 3.4.1. Overview

Cross-scale fusion combines high-level semantics with high-resolution spatial detail. From the perspective of a multi-scale feature hierarchy, this stage is critical because the high-resolution levels preserve weak local boundaries and compact component structures, whereas the deeper low-resolution levels provide semantic context. In degraded household scenes, however, cross-scale fusion may dilute weak high-resolution evidence, and the resulting encoder tokens may receive insufficient confidence during query selection. Therefore, DDRF is designed to enhance the hierarchy before candidate selection: CDRU restores detail-aware semantic information from the deep branch, DAFSE reinforces frequency-structural cues in the high-resolution branch, and the spatial gate adaptively suppresses unreliable enhanced responses caused by reflection, stains, or wall textures.

Blur, stains, dust, wall textures, metallic reflection, and viewpoint variation particularly affect small or boundary-sensitive structures, including In_needle, Ft_protection, Pipe_clamp, and local connection regions.

To alleviate these issues, DDRF is introduced into the cross-scale feature fusion stage of RT-DETR. Let $F_{i+1}$ denote the deep low-resolution semantic feature and $F_{i}$ denote the high-resolution feature at the current fusion level. Unlike conventional interpolation-and-concatenation fusion, DDRF progressively enhances and adaptively aggregates multi-scale features through detail recovery upsampling, frequency-structural enhancement, and spatial gated fusion. Specifically, $F_{i+1}$ is first processed by CDRU to generate the detail-recovered feature $F_{rec}^{i}$, while $F_{i}$ is enhanced by the DAFSE branch to produce the degradation-aware frequency-structural feature $F_{freq}^{i}$. The two features are then fused by a degradation-aware spatial gating mechanism to obtain $F_{out}^{i}$. This process can be summarized as

(1) $$F_{rec}^{i}=CDRU(F_{i+1}),$$

where $CDRU\left(\cdot \right)$ and $DAFSE\left(\cdot \right)$ denote the detail-recovery and frequency-structural branches, respectively. DDRF operates only in the feature-fusion path; the decoder and detection heads remain unchanged.

#### 3.4.2. Converse2D-Based Detail Recovery Upsampling Unit

**Figure 4:**
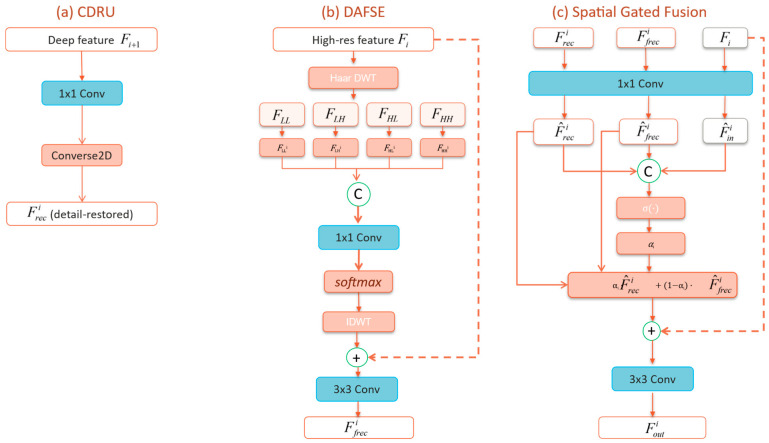
Structure of DDRF: (**a**) Converse2D-based detail-recovery upsampling; (**b**) degradation-aware frequency-structural enhancement; and (**c**) spatial gated fusion.

CDRU first aligns the channels of the deep low-resolution feature with a 1 × 1 convolution and then applies Converse2D for learnable detail-recovery upsampling (Figure 4a). Unlike the complete ConverseNet restoration network, this feature-level unit requires neither image reconstruction nor additional restoration supervision [34].

For a deep low-resolution feature, CDRU is formulated as follows:

(2) $$F_{i+1}\in R^{C_{i+1}\times \frac{H}{s}\times \frac{W}{s}}.$$

CDRU first uses a 1×1 convolution to map its channel number to the dimension required by the current fusion level:

(3) $$F_{align}^{i+1}=Conv_{1\times 1}(F_{i+1}),$$

where:

(4) $$F_{align}^{i+1}\in R^{C_{i}\times \frac{H}{s}\times \frac{W}{s}}.$$

Then, Converse2D with scale factor s is used for detail-recovery upsampling:

(5) $$F_{rec}^{i}=Converse2D_{s}(F_{align}^{i+1}),$$

where

(6) $$F_{rec}^{i}\in R^{C_{i}\times H\times W}.$$

Here, s is the stride between adjacent feature levels, and $F_{rec}^{i}$ is aligned with $F_{i}$ in spatial and channel dimensions. The recovered feature supplies structure-aware semantics for subsequent fusion.

#### 3.4.3. Degradation-Aware Frequency-Structural Enhancement Branch

DAFSE decomposes the current high-resolution feature with a channel-wise Haar DWT, enhances and reweights the four frequency bands, and reconstructs the result through IDWT with a residual spatial path (Figure 4b).

For the current-scale feature, the channel-wise Haar decomposition is defined as

(7) $$F_{i}\in R^{C_{i}\times H_{i}\times W_{i}}.$$

The transform produces one low-frequency approximation band and three directional high-frequency detail bands [14]:

(8) $$\left[F_{LL}^{i},F_{LH}^{i},F_{HL}^{i},F_{HH}^{i}\right]=DWT\left(F_{i}\right),$$

where each frequency band has the spatial size:

(9) $$F_{b}^{i}\in R^{C_{i}\times H_{i}/2\times W_{i}/2},\;b\in \{LL,LH,HL,HH\}.$$

Here, $F_{LL}^{i}$ preserves low-frequency structure, whereas $F_{LH}^{i}$, $F_{HL}^{i}$, and $F_{HH}^{i}$ encode directional high-frequency details.

Then, each frequency band $F_{b}^{i}$ is modeled by a lightweight band-enhancement operator $\phi_{b}\left(\cdot \right)$ composed of depth-wise convolution and 1×1 convolution:

(10) $$E_{b}^{i}=\phi_{b}\left(F_{b}^{i}\right),\;b\in \{LL,LH,HL,HH\},$$

where $\phi_{b}\left(\cdot \right)$ combines depth-wise and 1×1 convolutions, and $E_{b}^{i}$ is the enhanced feature of the b-th band.

The enhanced bands are concatenated so that spatially adaptive weights can be estimated for each frequency component:

(11) $$E_{cat}^{i}=Concat\left(E_{LL}^{i},E_{LH}^{i},E_{HL}^{i},E_{HH}^{i}\right).$$

Then, a 1×1 convolution is used to generate spatially adaptive weights along the band dimension, followed by a Softmax operation over the four bands at each spatial position:

(12) $$\left[A_{LL}^{i},A_{LH}^{i},A_{HL}^{i},A_{HH}^{i}\right]={Softmax}_{band}\left({Conv}_{1\times 1}\left(E_{cat}^{i}\right)\right),$$

where

(13) $$A_{b}^{i}\in R^{1\times H_{i}/2\times W_{i}/2},\;b\in \{LL,LH,HL,HH\}.$$

$A_{b}^{i}$ is the spatial weight of the b-th band, enabling local reweighting of structural and detail responses.

The enhanced frequency-band features are then recalibrated using the band weights:

(14) $${\hat{E}}_{b}^{i}=A_{b}^{i}⊙E_{b}^{i},\;b\in \left\{LL,LH,HL,HH\right\},$$

where ⊙ denotes element-wise multiplication, and the weight $A_{b}^{i}$ is broadcast along the channel dimension. After frequency-band reweighting, inverse wavelet transform reconstructs the multi-band feature back to the spatial domain:

(15) $$F_{mf}^{i}=IDWT\left({\hat{E}}_{LL}^{i},{\hat{E}}_{LH}^{i},{\hat{E}}_{HL}^{i},{\hat{E}}_{HH}^{i}\right),$$

where $F_{mf}^{i}$ denotes the spatial feature reconstructed from multiple frequency bands. To prevent frequency-domain processing from weakening the original spatial information, a residual structure-preserving path is introduced:

(16) $$F_{freq}^{i}=Conv_{3\times 3}\left(F_{mf}^{i}+Conv_{1\times 1}(F_{i})\right),$$

where $Conv_{1\times 1}\left(F_{i}\right)$ preserves the original spatial information in the current-scale feature and aligns it with the reconstructed multi-band feature in channel dimension. The final 3×3 convolution further integrates local responses after frequency reconstruction to obtain the final frequency-structural enhanced feature:

(17) $$F_{freq}^{i}\in R^{C_{i}\times H\times W}.$$

The reconstructed frequency-structural feature is subsequently passed to the spatial gate.

#### 3.4.4. Degradation-Aware Spatial Gated Fusion

The spatial gate combines the CDRU and DAFSE outputs while retaining the original current-scale feature (Figure 4c).

Specifically, given the detail-recovered feature $F_{rec}^{i}$ from CDRU, the frequency-structural enhanced feature $F_{freq}^{i}$ from DAFSE, and the original current-scale feature $F_{i}$, three 1×1 convolutions are first used for channel alignment and lightweight channel interaction:

(18) $${\hat{F}}_{rec}^{i}=Conv_{1\times 1}(F_{rec}^{i}),$$

where ${\hat{F}}_{rec}^{i}$, ${\hat{F}}_{freq}^{i}$, and ${\hat{F}}_{in}^{i}$ have the same spatial resolution and channel dimension. The three features are then concatenated along the channel dimension, and a 1×1 convolution followed by a Sigmoid function generates a spatially adaptive fusion weight:

(19) $$\alpha^{i}=\sigma \left(Conv_{1\times 1}\left(Concat({\hat{F}}_{rec}^{i},{\hat{F}}_{freq}^{i},{\hat{F}}_{in}^{i})\right)\right),$$

where $\sigma \left(\cdot \right)$ is the Sigmoid function and $\alpha^{i}$ is learned under detection supervision. Larger $\alpha^{i}$ emphasizes $F_{rec}^{i}$, whereas larger $1-\alpha^{i}$ emphasizes $F_{freq}^{i}$.

Based on this spatial weight, the recovered semantic feature and the frequency-structural feature are adaptively fused as

(20) $$F_{gate}^{i}=\alpha^{i}⊙{\hat{F}}_{rec}^{i}+(1-\alpha^{i})⊙{\hat{F}}_{freq}^{i},$$

where ⊙ denotes element-wise multiplication. Compared with fixed addition or simple concatenation, this gated fusion strategy dynamically adjusts information sources according to local feature quality, thereby reducing the influence of unreliable enhanced responses on subsequent detection.

Finally, to prevent the enhanced feature from completely overriding the original current-scale information, a residual feature-preserving path is introduced, and a 3×3 convolution is used to re-integrate local context after fusion:

(21) $$F_{out}^{i}=Conv_{3\times 3}\left(F_{gate}^{i}+{\hat{F}}_{in}^{i}\right).$$

The learned gate determines the local contribution of the recovered semantic and frequency-structural branches; the residual path preserves the original current-scale information.

DDRF uses only lightweight convolutions, DWT/IDWT, and spatial gating. It introduces neither expert routing nor an independent restoration network, thereby limiting the computational increase while improving degradation-affected feature representation.

### 3.5. Small-Object Guided Query Selection

**Figure 5:**
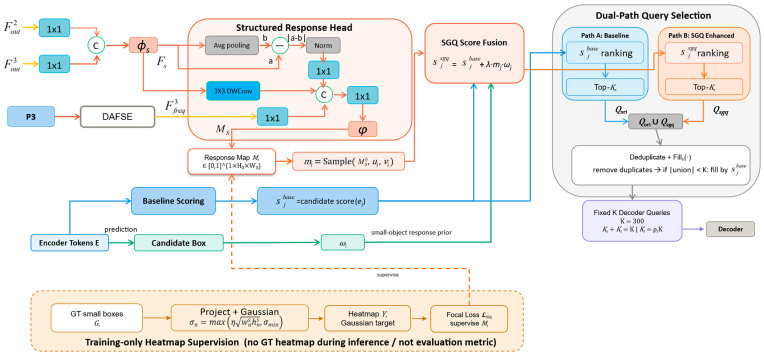
Small-object guided query selection pipeline. The block labeled “Norm” denotes batch normalization (BN). Solid colored arrows indicate different information paths for candidate scoring, small-object prior fusion, and query selection, whereas dashed arrows indicate training-only heatmap supervision.

Figure 5 summarizes SGQ, which converts DDRF-enhanced high-resolution features into a compact-object response prior and combines this prior with the baseline candidate score and scale modulation for dual-path selection under a fixed query budget. In relation to the multi-scale hierarchy discussion above, SGQ serves as the bridge between enhanced hierarchical representation and transformer query allocation. The enhanced high-resolution response is not appended as a long transformer sequence; instead, it is converted into a compact spatial prior that re-ranks encoder candidates. This design follows the insight that hierarchical local-global information should improve query initialization or candidate admission, while avoiding excessive encoder-token expansion. Consequently, SGQ improves small-object accessibility under the unchanged K = 300 query budget.

Because RT-DETR retains only the top-ranked encoder tokens, weak-response compact objects may remain unmodeled even after feature enhancement. SGQ addresses this limitation by re-ranking candidates with a lightweight spatial prior rather than increasing the decoder-query budget.

#### 3.5.1. DDRF-Coupled Structural Small-Object Response Map Generation

To avoid over-reliance on a fixed feature level, $F_{s}$ is defined as the highest-resolution DDRF-enhanced feature used to generate the small-object spatial prior. Let $F_{out}^{l_{s}}$ denote the highest-resolution level among the DDRF outputs. By default:

(22) $$F_{s}={Conv}_{1\times 1}(F_{out}^{l_{s}}),$$

where $l_{s}$ denotes the highest-resolution level used for small-object response map generation. This definition enables SGQ to adapt to different RT-DETR feature-level configurations without depending on a fixed scale.

In the implemented SGQ branch, the DDRF-enhanced P2 feature is used only as a high-resolution detail cue for response-map generation; it is not passed to the hybrid encoder or decoder and therefore does not increase the transformer token sequence. After channel alignment, the enhanced P2 and P3 features are fused at P3 resolution.

(23) $${\tilde{F}}_{2}={Conv}_{1\times 1}(F_{out}^{2}),{\tilde{F}}_{3}={Conv}_{1\times 1}(F_{out}^{3}).$$

Then, ${\tilde{F}}_{2}$ is downsampled to the $P_{3}$ scale and lightly fused with ${\tilde{F}}_{3}$:

(24) $$F_{s}=\phi_{s}\left(Concat\left({Down}_{2}({\tilde{F}}_{2}),{\tilde{F}}_{3}\right)\right).$$

The fusion unit applies stride-2 downsampling to P2, followed by a 3 × 3 depth-wise convolution, a 1 × 1 point-wise convolution, Batch Normalization, and a nonlinear activation. This path contributes high-resolution detail cues exclusively to the response branch.

After obtaining $F_{s}$, a DDRF-coupled structured response branch is further constructed. Unlike a plain $Conv_{3\times 3}+Conv_{1\times 1}+Sigmoid$ response head, this branch jointly models local structural cues, local contrast cues, and frequency-structural cues, thereby more specifically highlighting weak-boundary small objects in degraded scenes.

First, depth-wise convolution is used to extract local structural cues:

(25) $$F_{loc}={DWConv}_{3\times 3}(F_{s}).$$

The local structural feature $F_{loc}$ captures compact local structures around small objects, such as fine local boundaries, curved component contours, and reflective connection edges. Because depth-wise convolution is computationally lightweight, this branch enhances local structural responses of small objects at limited cost.

A local-contrast cue is also introduced to suppress wall textures, stains, and metallic reflections that can produce high responses unrelated to true compact objects:

(26) $$F_{con}={Conv}_{1\times 1}\left(Norm\left(\left|F_{s}-{AvgPool}_{k\times k}(F_{s})\right|\right)\right),$$

where AvgPool_(k×k) (·) computes the local mean and Norm(·) is implemented as Batch Normalization (BN). The normalized residual suppresses background-induced peaks while retaining locally distinctive responses supported by the feature semantics.

In addition, to couple SGQ more closely with DDRF, the frequency-structural enhanced feature already generated by the DAFSE branch in DDRF is reused. Let $F_{freq}^{3}$ denote the frequency-structural enhanced feature output by DAFSE at the $P_{3}$ level:

(27) $$F_{freq}^{3}=DAFSE(F_{3}).$$

Because $F_{freq}^{3}$ is already generated in DDRF, SGQ only applies channel projection to it and does not introduce a new frequency-domain decomposition branch:

(28) $$F_{str}={Conv}_{1\times 1}(F_{freq}^{3}).$$

In DDRF, $F_{freq}^{3}$ is used for structural enhancement during cross-scale fusion; in SGQ, it serves only as a structural prior for response map generation, indicating potential small-object candidate regions. The frequency-structural representation is therefore shared across representation enhancement and query initialization. In this way, $F_{str}$ provides the response branch with edge, texture, and local structural information from the frequency-structural enhancement path, helping the model focus on weak boundaries of real gas facility components rather than noise-like high-frequency background responses.

To control computational overhead, the three cues above are compressed to a small channel dimension before fusion:

(29) $$F_{loc},F_{con},F_{str}\in R^{C_{r}\times H_{3}\times W_{3}},C_{r}\ll C.$$

Then, the three cues are concatenated to generate a structured small-object representation:

(30) $$F_{srm}={Conv}_{1\times 1}\left(Concat(F_{loc},F_{con},F_{str})\right).$$

Finally, a 1×1 convolution followed by Sigmoid activation is used to generate the small-object response map:

(31) $$M_{s}=\sigma \left({Conv}_{1\times 1}(F_{srm})\right),$$

where

(32) $$M_{s}\in {\left[0,1\right]}^{1\times H_{3}\times W_{3}}.$$

$M_{s}$ is a query-selection prior rather than a segmentation mask; it uses existing box annotations for training and introduces no segmentation labels.

#### 3.5.2. Small-Object Heatmap Supervision

When $M_{s}$ is supervised only through the final detection loss, its response distribution may be unstable due to background textures, degradation noise, and class-confidence fluctuations. In particular, when small-object regions are sparse and positive/negative samples are highly imbalanced, a response branch without explicit supervision may fail to stably focus on real small-object regions. Therefore, existing bounding-box annotations are further used to generate small-object heatmaps for auxiliary supervision on $M_{s}$. This supervision is used only during training, and no ground-truth heatmap is required during inference.

The auxiliary object-selection rule is used solely to construct the training heatmap; it does not define a custom evaluation subset. All reported detection results follow the COCO-style protocol [35].

Given the ground-truth annotation set:

(33) $$G=\{g_{n}=(x_{n},y_{n},w_{n},h_{n},c_{n}){\}}_{n=1}^{N_{g}},$$

where $\left(x_{n},y_{n}\right)$ denotes the object center, $\left(w_{n},h_{n}\right)$ denotes the object width and height, and $c_{n}$ denotes the class label. Because object scales differ substantially across categories, the training-only heatmap target uses a combined global-area and within-class criterion:

(34) $$G_{s}=\left\{g_{n}\in G|\frac{w_{n}h_{n}}{HW}\leq \tau_{s}or\left(c_{n}\in C_{s}\wedge r_{n}\leq \tau_{s}^{\prime }\right)\right\}.$$

Here, H and W denote the input dimensions and the global normalized-area threshold is 0.030. The category-aware set contains In_needle and Ft_protection, the two small-object-dominant classes identified from the training statistics in Table 1. For these classes, the empirical within-class area percentile is computed from the training annotations, and the smallest 40% of instances are retained. These quantities are fixed before validation and testing.

This training-only rule does not alter the standard COCO small-object definition or any reported metric.

For each small object $g_{n}\in G_{s}$, its center is projected onto the $P_{3}$ feature scale to obtain $\left(x_{n}^{s},y_{n}^{s}\right)$. A Gaussian heatmap is then generated around this center:

(35) $$Y_{s}(u,v)=\underset{g_{n}\in G_{s}}{max}exp\left(-\frac{{\left(u-x_{n}^{s}{)}^{2}+(v-y_{n}^{s}\right)}^{2}}{2\sigma_{n}^{2}}\right),$$

where $\left(u,v\right)$ denotes a spatial position on the $P_{3}$ response map, and $\sigma_{n}$ is adaptively determined according to the projected object size:

(36) $$\sigma_{n}=max\left(\eta \sqrt{w_{n}^{s}h_{n}^{s}},\sigma_{min}\right),$$

where η=0.5 and $\sigma_{min}=1$.

The Gaussian coefficient is fixed at 0.50, and a minimum radius of one feature-map pixel prevents degenerate targets for extremely small projected boxes. Relative to a binary center label, the Gaussian target provides smoother supervision and reduces sensitivity to feature-map quantization.

The small-object response map $M_{s}$ is supervised using a focal-style heatmap loss [36]:

(37) $$L_{sgq}=-\frac{1}{H_{3}W_{3}}\sum_{u=1}^{H_{3}}\sum_{v=1}^{W_{3}}\left[\alpha Y_{s}\left(u,v\right){\left(1-M_{s}\left(u,v\right)\right)}^{\gamma_{hm}}logM_{s}\left(u,v\right)+\left(1-\alpha \right)\left(1-Y_{s}\left(u,v\right)\right)M_{s}{\left(u,v\right)}^{\gamma_{hm}}log\left(1-M_{s}\left(u,v\right)\right)\right],$$

where α balances positive and negative regions and $\gamma_{hm}$ downweights easy background positions. At inference, only the predicted $M_{s}$ is used.

#### 3.5.3. Dual-Path Query Selection Strategy

After the hybrid encoder, the multi-scale encoder features are flattened into a token sequence:

(38) $$E=\{e_{j}{\}}_{j=1}^{N}.$$

Each token $e_{j}$ has a baseline query selection score $s_{j}^{base}$ and an initial predicted box:

(39) $$b^{j}=\left(x^{j},y^{j},w^{j},h^{j}\right).$$

In the implemented RT-DETR baseline, the candidate score is the maximum class confidence predicted by the encoder classification head. SGQ changes only the ranking used for query selection and leaves the encoder classification and box-prediction heads unchanged.

To introduce the small-object spatial prior, $M_{s}$ is first resized to the spatial scale of each encoder feature level. For the j-th encoder token, with spatial position $\left(u_{j},v_{j}\right)$ and feature level $l_{j}$, the corresponding small-object response score is obtained by bilinear sampling:

(40) $$m_{j}=Sample(M_{s}^{l_{j}},u_{j},v_{j}),$$

where $M_{s}^{l_{j}}$ denotes the small-object response map resized to the scale of the $l_{j}$-th level. To avoid overly promoting large objects or broad background structures only because of high responses, a scale-aware modulation term is further introduced. The normalized area of the j-th candidate box is first defined as

(41) $$a_{j}=w^{j}h^{j},$$

where ${\hat{w}}_{j}$ and ${\hat{h}}_{j}$ denote the normalized predicted width and height. The scale-aware modulation term is defined as

(42) $$\omega_{j}=exp(-\frac{sg(a_{j})}{\tau_{a}}),$$

where $\tau_{a}$ controls scale modulation and $sg\left(\cdot \right)$ stops gradients through the candidate area. Thus, smaller boxes receive larger ranking compensation without driving the box branch to shrink predictions.

Combining the baseline score, small-object response score, and scale modulation term, the SGQ-enhanced candidate score is defined as:

(43) $$s_{j}^{sgq}=s_{j}^{base}+\lambda m_{j}\omega_{j},$$

where λ is the small-object guidance weight. This score introduces the small-object spatial prior from the DDRF-coupled response map while preserving the semantic confidence of baseline query selection.

Based on $s_{j}^{base}$ and $s_{j}^{sgq}$, a dual-path query selection strategy is adopted:

(44) $$Q_{ori}=TopK(\{s_{j}^{base}{\}}_{j=1}^{N},K_{o}),$$

where $K_{o}+K_{s}=K$, $K_{s}=\rho_{s}K$, and $\rho_{s}$ is the guided-query ratio. Duplicate spatial indices are removed, and unfilled slots are completed by descending $s_{j}^{base}$ so that the decoder always receives K queries.

### 3.6. Scale-Adaptive Geometric Refinement Loss

**Figure 6:**
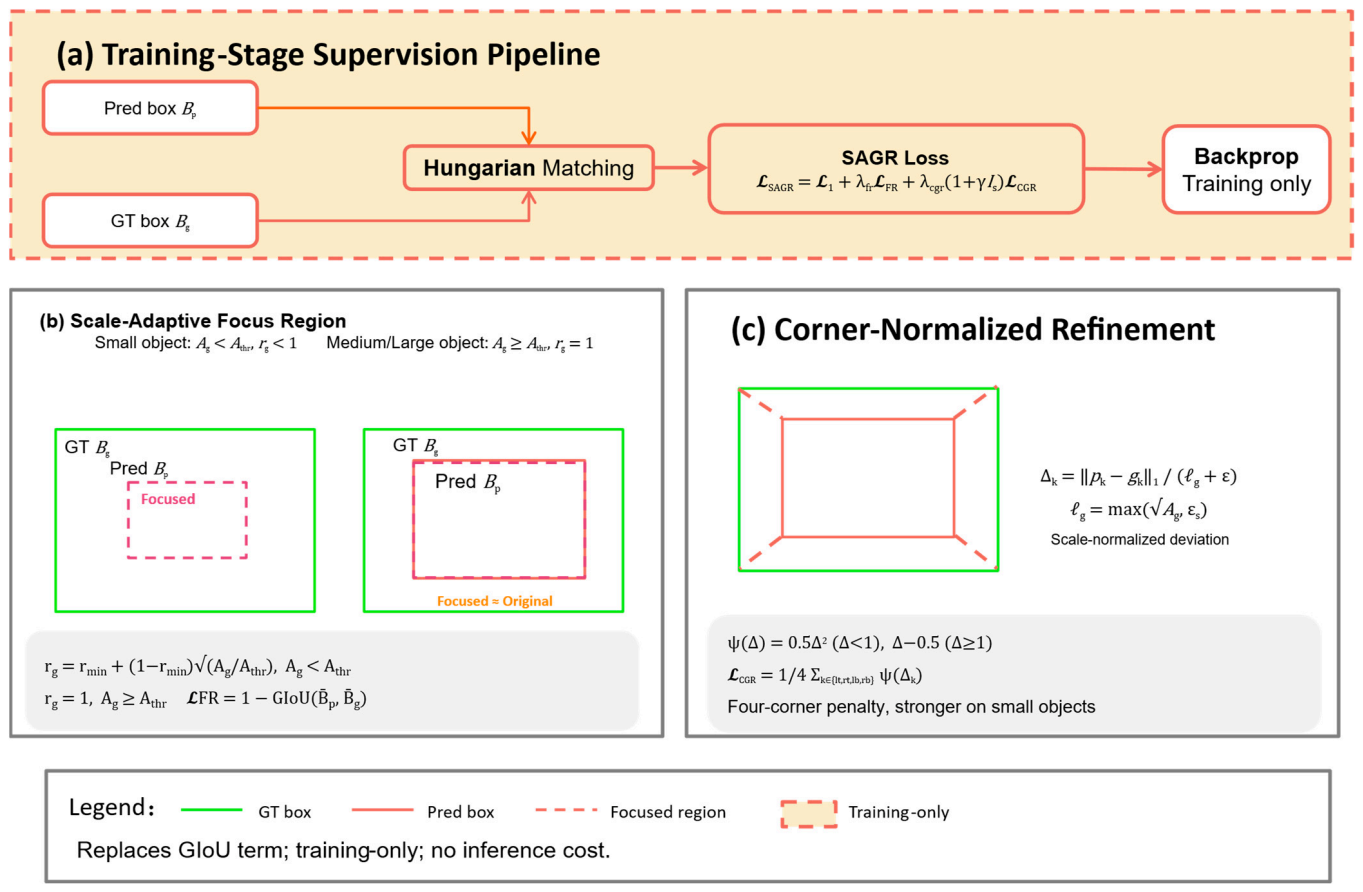
Scale-adaptive geometric refinement loss: (**a**) training-stage supervision; (**b**) scale-adaptive focused regions; and (**c**) corner-normalized refinement.

Figure 6 summarizes SAGR, the training-only box-regression objective for Hungarian-matched positive queries. It retains the L1 coordinate-regression term and replaces the original GIoU localization term with scale-adaptive focused-region overlap and corner-normalized geometric refinement, without changing the decoder or inference path.

#### 3.6.1. Scale-Adaptive Focus Region Construction

Figure 6b illustrates the focused-region construction. The predicted box and its Hungarian-matched ground-truth box are represented in normalized center format as

(45) $$B_{p}=\left(x_{p},y_{p},w_{p},h_{p}\right),\;B_{g}=\left(x_{g},y_{g},w_{g},h_{g}\right),$$

where $\left(x_{p},y_{p}\right)$ and $\left(x_{g},y_{g}\right)$ denote the centers of the predicted box and the ground-truth box, respectively, and $\left(w_{p},h_{p}\right)$ and $\left(w_{g},h_{g}\right)$ denote their widths and heights. Following the common implementation of RT-DETR, all box coordinates are normalized to the range $\left[0,1\right]$.

A fixed geometric constraint affects small and larger objects differently because the same absolute displacement represents a larger relative error for a compact target. The normalized ground-truth area is therefore used to determine the focusing ratio:

(46) $$A_{g}=w_{g}h_{g}.$$

Given the small-object area threshold $A_{thr}$, the scale-adaptive focusing ratio is defined as

(47) $$r_{g}=\left\{\begin{array}{ll} r_{min}+\left(1-r_{min}\right)\sqrt{\frac{A_{g}}{A_{thr}}}, & A_{g}<A_{thr},\\ 1, & A_{g}\geq A_{thr}, \end{array}\right.$$

where $r_{min}\in \left(0,1\right)$ is the minimum focusing ratio. For small $A_{g}$, $r_{g}$ approaches $r_{min}$; when $A_{g}$ ≥ $A_{thr}$, $r_{g}=1$.

Based on $r_{g}$, the scale-adaptive focusing regions of the predicted and ground-truth boxes are defined as:

(48) $${\bar{B}}_{p}=\left(x_{p},y_{p},r_{g}w_{p},r_{g}h_{p}\right),$$

(49) $${\bar{B}}_{g}=\left(x_{g},y_{g},r_{g}w_{g},r_{g}h_{g}\right).$$

These are loss-computation regions rather than new predictions. For medium or large objects, $r_{g}=1$, recovering the original box geometry.

The focusing-region overlap loss is defined as

(50) $$L_{FR}=1-GIoU\left({\bar{B}}_{p},{\bar{B}}_{g}\right).$$

GIoU is used for focusing-region overlap because it provides gradients for non-overlapping boxes; ϵ is added only for numerical stability.

#### 3.6.2. Corner-Normalized Geometric Refinement

Corner-normalized refinement distinguishes boxes that have similar overlap but different boundary positions or aspect ratios (Figure 6c). Because the annotations are horizontal boxes, the four corners can be constrained without additional contour labels.

The predicted and ground-truth boxes are converted into four-corner representations:

(51) $$B_{p}=\left(p_{lt},p_{rt},p_{lb},p_{rb}\right),$$

where lt, rt, lb, and rb denote the left-top, right-top, left-bottom, and right-bottom corners, respectively. For any corner k∈{lt,rt,lb,rb}, the scale-normalized corner offset is defined as

(52) $$\Delta_{k}=\frac{∥p_{k}-g_{k}{∥}_{1}}{l_{g}+\epsilon },$$

where ϵ is a small constant for numerical stability, and $l_{g}$ denotes the characteristic scale of the target. To avoid excessively large normalized corner errors for extremely small objects, a minimum scale bound is applied to $l_{g}$ in implementation:

(53) $$l_{g}=max\left(\sqrt{A_{g}},\epsilon_{s}\right),$$

where $\epsilon_{s}$ is the minimum scale threshold. Normalization by $l_{g}$ gives the same absolute corner error, with a larger penalty for smaller objects.

To avoid excessively large gradients from hard samples in early training, a smooth geometric penalty function is used:

(54) $$\psi (\Delta_{k})=\{\begin{array}{cc} 0.5\;\Delta_{k}^{2}, & \Delta_{k}<1,\\ \Delta_{k}-0.5, & \Delta_{k}\geq 1. \end{array}$$

The corner-normalized geometric refinement loss is then obtained as

(55) $$L_{CGR}=\frac{1}{4}\sum_{k\in \{lt,rt,lb,rb\}}\;\psi (\Delta_{k}).$$

By penalizing all four scale-normalized corner offsets, this term jointly constrains position, width, height, and shape alignment, which is particularly relevant to boundary-sensitive compact components.

To maintain consistency with the training-stage small-object supervision rule defined in Section 3.5.2, the small-object indicator is defined as

(56) $$I_{s}=\{\begin{array}{cc} 1, & B_{g}\in G_{s},\\ 0, & B_{g}\notin G_{s}, \end{array}$$

where $G_{s}$ denotes the training-stage small-object supervision set defined in Section 3.5.2. The final SAGR loss is defined as

(57) $$L_{SAGR}=L_{1}+\lambda_{fr}L_{FR}+\lambda_{cgr}\left(1+\gamma I_{s}\right)L_{CGR}.$$

In Equation (57), L1 is the baseline coordinate-regression term; the focused-region and corner-refinement weights balance the two geometric components, and the small-object factor increases the penalty for compact targets. The final values are listed in Section 3.8.

Thus, the complete box-regression objective retains the baseline L1 coordinate-regression term and replaces the original GIoU localization term with the SAGR formulation defined in Equation (57). It is evaluated only for Hungarian-matched positive queries during training; none of its intermediate quantities are computed at inference.

### 3.7. Training Objective and Inference Procedure

**Figure 7:**
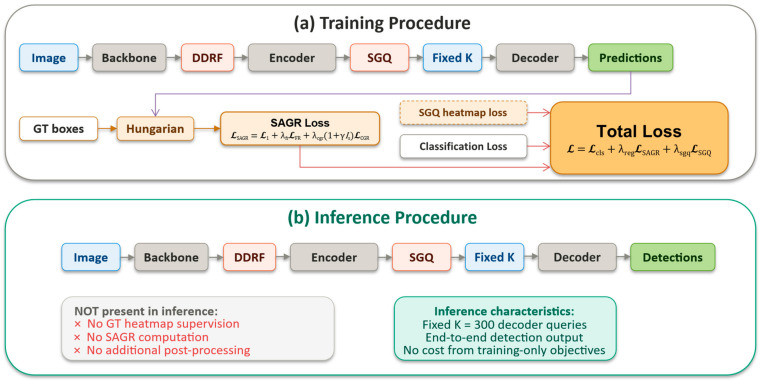
Training and inference paths of DRQ-RTDETR. SAGR and SGQ heatmap supervision are used only during training; DDRF and SGQ query selection remain active during inference.

Figure 7 distinguishes the training and inference paths. Training combines baseline classification, the SAGR box-regression objective, and auxiliary SGQ heatmap supervision. At inference, the two training-only objectives are removed, while DDRF and SGQ query selection remain active and account for the measured runtime overhead.

Hungarian matching assigns queries to targets during training. Matched positive boxes are optimized with SAGR, and the SGQ response map receives auxiliary heatmap supervision. At inference, these loss computations are removed, whereas DDRF and SGQ remain in the forward path and account for the additional parameters, FLOPs, and FPS reduction reported in the experimental results.

The overall training objective is therefore defined as

(58) $$L_{total}=L_{cls}+\lambda_{reg}L_{SAGR}+\lambda_{sgq}L_{SGQ},$$

where *λ_reg_* weights the SAGR box-regression objective defined in Equation (57), and *λ_sgq_* weights the SGQ auxiliary heatmap term.

### 3.8. Implementation Details and Hyperparameter Protocol

The implemented SGQ configuration was fixed as follows: small-object guidance weight λ = 0.35, dual-path query ratio ρs = 0.30, scale-modulation temperature τa = 0.50, Gaussian coefficient η = 0.50, minimum Gaussian radius σmin = 1, and auxiliary heatmap-loss weight λsgq = 1.0. The response map is generated at P3 resolution by fusing the DDRF-enhanced P3 feature with a stride-2-downsampled P2 detail feature; Batch Normalization is used in the local-contrast branch, and the baseline query score is the maximum encoder class confidence. The training-only small-object rule uses τs = 0.030, Cs = {In_needle, Ft_protection}, and τs′ = 0.40. The SAGR parameters are rmin = 0.50, λfr = 1.0, λcgr = 0.50, and γ = 1.5.

The SGQ and SAGR hyperparameters were selected on a held-out tuning subset comprising 10% of the training images before final validation evaluation. The final values were fixed for all detector comparisons and ablation studies. A one-factor-at-a-time sensitivity analysis around the selected values is reported in Section 4.3.5 to verify that the selected settings represent stable operating points rather than narrow optima.

All models were trained using stochastic gradient descent (SGD) with a momentum of 0.937, a weight decay of 5 × 10^−4^, and Nesterov momentum enabled. A linear warm-up phase of 1000 iterations was applied, during which the learning rate increased from 1 × 10^−4^ to the initial value of 0.01, followed by a linear decay to 1 × 10^−4^ over the remaining epochs. All models were initialized from the official RT-DETR-R18vd pretrained COCO weights (release v1.0; file rtdetr_r18vd_dec_3x_coco.pth).

The random seed was set for Python’s random module, NumPy, PyTorch CPU and CUDA RNGs, and the DataLoader worker initialization. Each configuration was trained three times with seeds 42, 123, and 2024 to produce independent replicates. The software environment was Python 3.10.13, PyTorch 2.1.2 with CUDA 12.1 and cuDNN 8.9.2, torchvision 0.16.2, OpenCV 4.9.0, and NumPy 1.26.2. Training and inference were conducted on a single NVIDIA GeForce RTX 4060 Laptop GPU with 8 GB VRAM under Windows 11. The training and reproducibility settings used for all experiments are summarized in Table 2.

The data augmentation pipeline was applied in the following order: random horizontal flip with probability 0.5; random scaling with a factor sampled uniformly from [0.5, 1.5]; random translation with a ratio sampled uniformly from [−0.1, 0.1] in both x and y; color jitter with brightness, contrast, and saturation factors sampled from [0.8, 1.2] and hue from [−0.1, 0.1]; and mild affine transformation with rotation from [−5°, 5°] and shear from [−0.05, 0.05]. Mosaic and mixup were not used, and test-time augmentation was not applied.

Run-to-run variation was handled by training the principal configurations with three independent seeds and reporting mean ± standard deviation in the comparison and key ablation tables. The evaluation protocol and paired statistical test are described in Section 4.1.

Parameter counts and FLOPs were measured at 640 × 640 input resolution. FPS was measured on the same hardware under identical inference settings for all models.

FPS was measured on the same NVIDIA GeForce RTX 4060 Laptop GPU for all models using input resolution 640 × 640, batch size 1, FP32 precision, 50 warm-up iterations, and 200 timed iterations. The reported FPS includes image loading, resizing, model inference, and post-processing; for DETR-based detectors, the score-filtering step at confidence threshold 0.001 is included and non-maximum suppression is not required. CUDA synchronization was enforced after each iteration using torch.cuda.synchronize().

## 4. Results

The experiments evaluate overall accuracy, class- and degradation-specific behavior, staged module contributions, qualitative responses, failure modes, and computational efficiency. All analyses use the fixed scene-level split described in Section 3.1.

### 4.1. Evaluation Protocol and Statistical Reporting

Performance is reported using COCO-style mAP@[0.50:0.95], mAP50, mAP75, and mAPsmall. Class-wise AP50 and class-wise COCO-style AP are listed separately to distinguish category-level recognition from aggregate detector performance. The implementation and training settings are reported once in Section 3.8.

All accuracy metrics are reported as mean ± standard deviation over three independent training runs with random seeds 42, 123, and 2024. For the principal comparison between RT-DETR and DRQ-RTDETR, seed-matched paired differences were computed for each metric, and a two-sided paired *t*-test was applied. The 95% confidence interval (CI) of the paired difference and the corresponding *p*-value are reported in Table 3. External baseline detectors were re-trained under the same data split and settings; their mean ± SD values are reported alongside DRQ-RTDETR.

### 4.2. Comparison with State-of-the-Art Detectors

Table 3 compares DRQ-RTDETR with representative two-stage, CNN-based, and DETR-based detectors, including Faster R-CNN [37], YOLOv8-s [38], YOLOv10-s [39], DINO [11], and RT-DETR [3], under the same 640 × 640 evaluation setting. The comparison includes COCO-style accuracy, small-object performance, model size, computational cost, and throughput so that accuracy gains are interpreted together with deployment overhead rather than in isolation.

#### 4.2.1. Accuracy–Efficiency Trade-Off

Relative to RT-DETR, DRQ-RTDETR improves mAP by 4.13 percentage points, mAP75 by 4.34 points, and mAPsmall by 6.15 points, with 2.6 M additional parameters (13.1%), 6.4 G additional FLOPs (11.1%), and a 1.0 FPS throughput reduction (1.3%). The larger gain in mAPsmall is consistent with the intended emphasis on compact weak-response components. DRQ-RTDETR also achieves higher accuracy than DINO with substantially fewer parameters and FLOPs, whereas YOLOv8-s and YOLOv10-s remain faster but less accurate on small objects. These results indicate a task-specific accuracy-efficiency trade-off rather than uniform superiority.

The throughput results were measured under the unified FPS protocol described in Section 3.8; therefore, the accuracy-efficiency interpretation compares detectors under the same input size, numerical precision, batch size, and preprocessing/post-processing settings.

#### 4.2.2. Class-Wise Performance and Error Analysis

**Figure 8:**
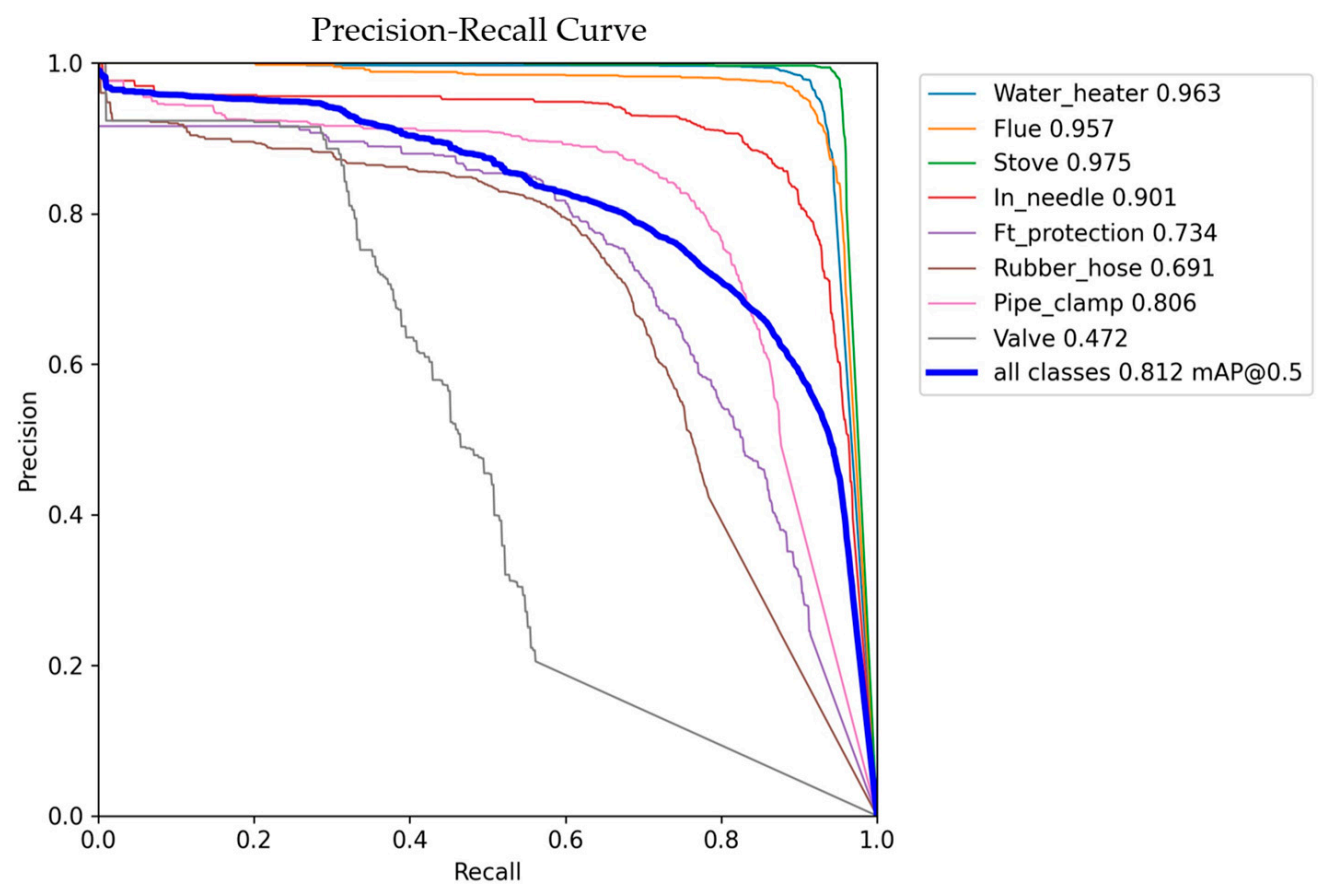
Precision–recall curves, class-wise AP_50_ values, and overall mAP_50_ of DRQ-RTDETR on the updated 8-class validation set.

Table 4 and Figure 8 show the strongest class-wise performance for Water_heater, Flue, Stove, and In_needle, whereas Valve, Rubber_hose, and Ft_protection remain more difficult. Valve is rare and has a compact, context-dependent appearance; Rubber_hose and Ft_protection are affected by flexible geometry, weak boundaries, partial occlusion, and similarity to neighboring pipes or wall textures. Residual errors are therefore concentrated in rare, deformable, or weakly separated categories.

**Figure 9:**
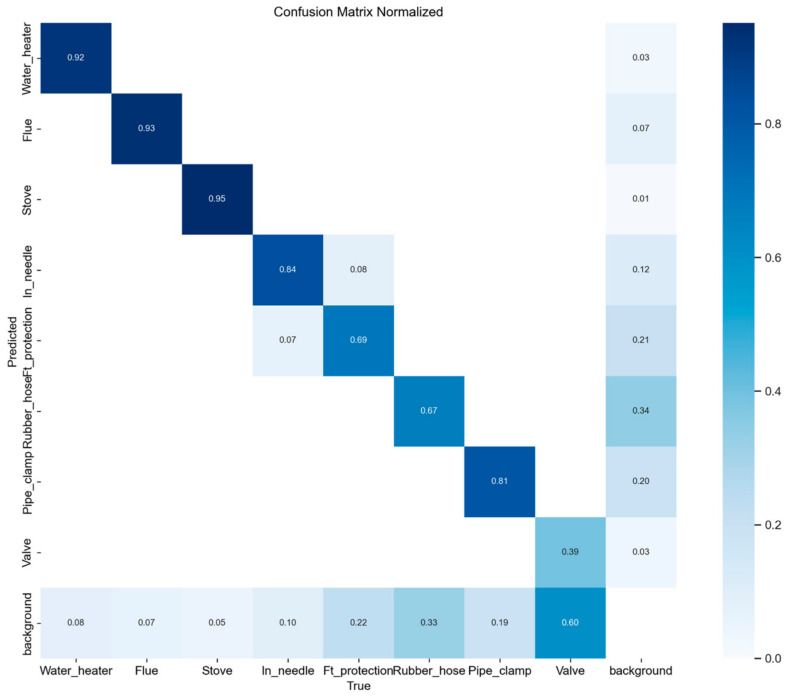
Normalized confusion matrix of DRQ-RTDETR on the updated 8-class validation set.

The normalized confusion matrix in Figure 9 supports this interpretation: dominant categories show stronger diagonal responses, whereas the difficult categories exhibit greater background confusion and cross-class ambiguity. Because normalization can conceal class-frequency effects, Figure 9 is interpreted together with the class-level instance distribution reported in Table 1.

The PR curves and confusion matrix identify Valve, Rubber_hose, and Ft_protection as the principal sources of residual error, motivating the failure-case analysis and the need for improved rare-class coverage, flexible-shape modeling, and suppression of high-frequency background interference.

For readability, Figure 8 and Figure 9 were prepared with enlarged labels, consistent class names, and publication-width legends; the confusion-matrix values are row-normalized to show the distribution of predictions for each ground-truth category.

#### 4.2.3. Robustness Under Degradation Conditions

To evaluate robustness across degradation conditions, the fixed validation set was stratified according to the image-level degradation labels defined in Section 3.1. For each degradation type, mAP and mAPsmall are reported as image-count-weighted averages over the mild, moderate, and severe subsets. The degradation-stratified performance comparison on the fixed validation set is summarized in Table 5.

### 4.3. Ablation Studies and Mechanistic Attribution

The staged ablations test whether the observed gains align with the intended function of each component. Accordingly, mAPsmall is emphasized for DDRF and SGQ, while mAP75 is emphasized for SAGR, alongside overall mAP.

#### 4.3.1. Overall Contributions of DDRF, SGQ, and SAGR

All values in Table 6 are reported as mean ± standard deviation over the three independent seeds. Seed-matched improvements are included to clarify the incremental contributions of DDRF, SGQ, and SAGR.

Table 6 shows monotonic improvement across the three intervention stages. DDRF increases mAPsmall by 2.59 percentage points and mAP75 by 2.14 points over RT-DETR. SGQ contributes a further 2.35-point gain in mAPsmall without increasing the decoder-query budget, and SAGR adds 0.94 points in mAP75 and 1.21 points in mAPsmall over DDRF + SGQ. The full 6.15-point mAPsmall gain is therefore consistent with complementary rather than redundant effects.

#### 4.3.2. DDRF Component Analysis

Table 7 separates detail-recovery upsampling, frequency-structural enhancement, and spatial gated fusion to determine whether the DDRF gain arises from a single operator or their coordinated use.

CDRU and DAFSE each improve the baseline independently, with DAFSE producing the larger single-branch gain. Their combination improves all accuracy metrics, and spatial gating yields the complete DDRF result of 0.6358 mAP and 0.4891 mAPsmall. The progression supports complementary detail recovery, frequency-structural enhancement, and adaptive fusion. The throughput change from 76.2 to 74.1 FPS should be interpreted under the unified FPS protocol described in Section 3.8. The effect of different upsampling variants in DDRF is summarized in Table 8.

Nearest-neighbor and bilinear interpolation yield the lowest accuracy, transposed convolution provides an intermediate result, and Converse2D/CDRU achieves the highest mAP, mAPsmall, and mAP75. With the remaining DDRF components fixed, the comparison attributes the improvement more directly to learnable detail-recovery upsampling.

#### 4.3.3. SGQ Component Analysis

Table 9 evaluates the response map, heatmap supervision, scale modulation, and dual-path selection cumulatively while retaining K = 300 decoder queries, thereby separating improved query allocation from increased query capacity.

The response map provides the first mAPsmall improvement, and explicit heatmap supervision yields a further gain, indicating that direct spatial supervision stabilizes the prior. Scale modulation and dual-path selection then raise mAPsmall to 0.5126 without increasing K. The cumulative 2.35-point gain over DDRF alone is therefore consistent with improved access to weak-response compact candidates rather than a larger decoder.

#### 4.3.4. SAGR Localization Analysis

Table 10 compares box-regression objectives under the same detector configuration. L1 is retained in every setting, and SAGR is evaluated as a scale-adaptive replacement for the original GIoU localization term while retaining the L1 coordinate-regression term. SAGR is applied only to Hungarian-matched positive queries during training and adds no inference branch.

All alternative objectives improve on the L1 + GIoU reference, with SAGR achieving the highest mAP (0.6576), mAP75 (0.7136), and mAPsmall (0.5247). The larger gains at strict IoU and for compact objects are consistent with focused-region overlap and corner-normalized refinement. Relative to L1 + MPDIoU, SAGR improves mAP75 by 0.29 percentage points and mAPsmall by 0.41 points.

#### 4.3.5. Hyperparameter Sensitivity and Run-to-Run Stability

A one-factor-at-a-time sensitivity analysis was conducted for the five principal SGQ and SAGR parameters. Each parameter was varied around the selected value while the others were fixed; each setting was trained with three seeds. Table 11 summarizes the selected value, tested range, and observed metric range for each principal SGQ and SAGR parameter.

The selected values are not narrow optima: moderate perturbations lead to gradual performance changes rather than abrupt performance degradation. The largest sensitivity appears in mAPsmall for λ and ρs, matching their role in small-object query allocation, while rmin and γ affect mAP75 more directly because they control boundary-sensitive localization.

### 4.4. Qualitative Comparison and Response Visualization

Qualitative analysis examines whether the quantitative gains correspond to improved localization and response concentration in representative household scenes. Such evidence complements, but does not replace, controlled ablation and statistical analysis.

**Figure 10:**
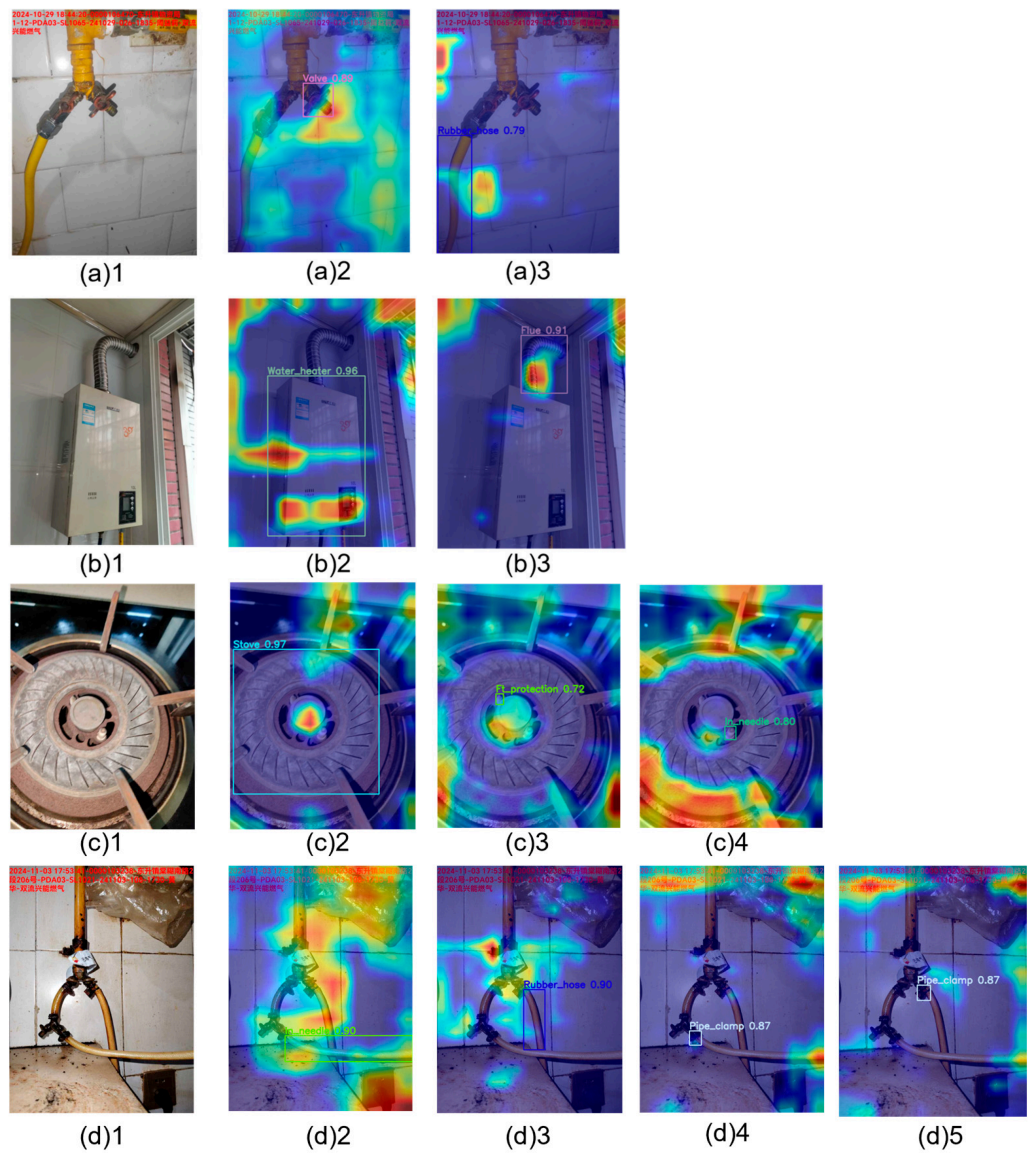
Qualitative detection and response-heatmap visualization under complex household gas-facility scenes. Visible non-English characters and top metadata, if present, are incidental acquisition information from the original inspection images and are not used for class annotation, model prediction, or performance evaluation. (**a1**) Original valve/pipe connection scene; (**a2**) response visualization and detection result for the valve region; (**a3**) response visualization and detection result for the rubber-hose region; (**b1**) original water-heater scene; (**b2**) response visualization and detection result for the water-heater region; (**b3**) response visualization and detection result for the flue region; (**c1**) original stove scene; (**c2**) response visualization and detection result for the stove region; (**c3**) response visualization and detection result for the Ft_protection region; (**c4**) response visualization and detection result for the In_needle region; (**d1**) original complex connection scene with hose and pipe components; (**d2**) response visualization and detection result for the In_needle region; (**d3**) response visualization and detection result for the rubber-hose region; (**d4**) response visualization and detection result for a pipe-clamp region; and (**d5**) response visualization and detection result for another pipe-clamp region.

The examples in Figure 10 cover connection structures, rubber hoses, pipe clamps, valves, stove-related components, In_needle, and Ft_protection. When aligned with the original image and both detector outputs, the response maps can show whether DRQ-RTDETR suppresses wall clutter and concentrates on compact boundaries under reflection, partial occlusion, and low contrast. Heatmaps alone should not be interpreted as causal evidence.

### 4.5. Failure Cases and Practical Limitations

**Figure 11:**
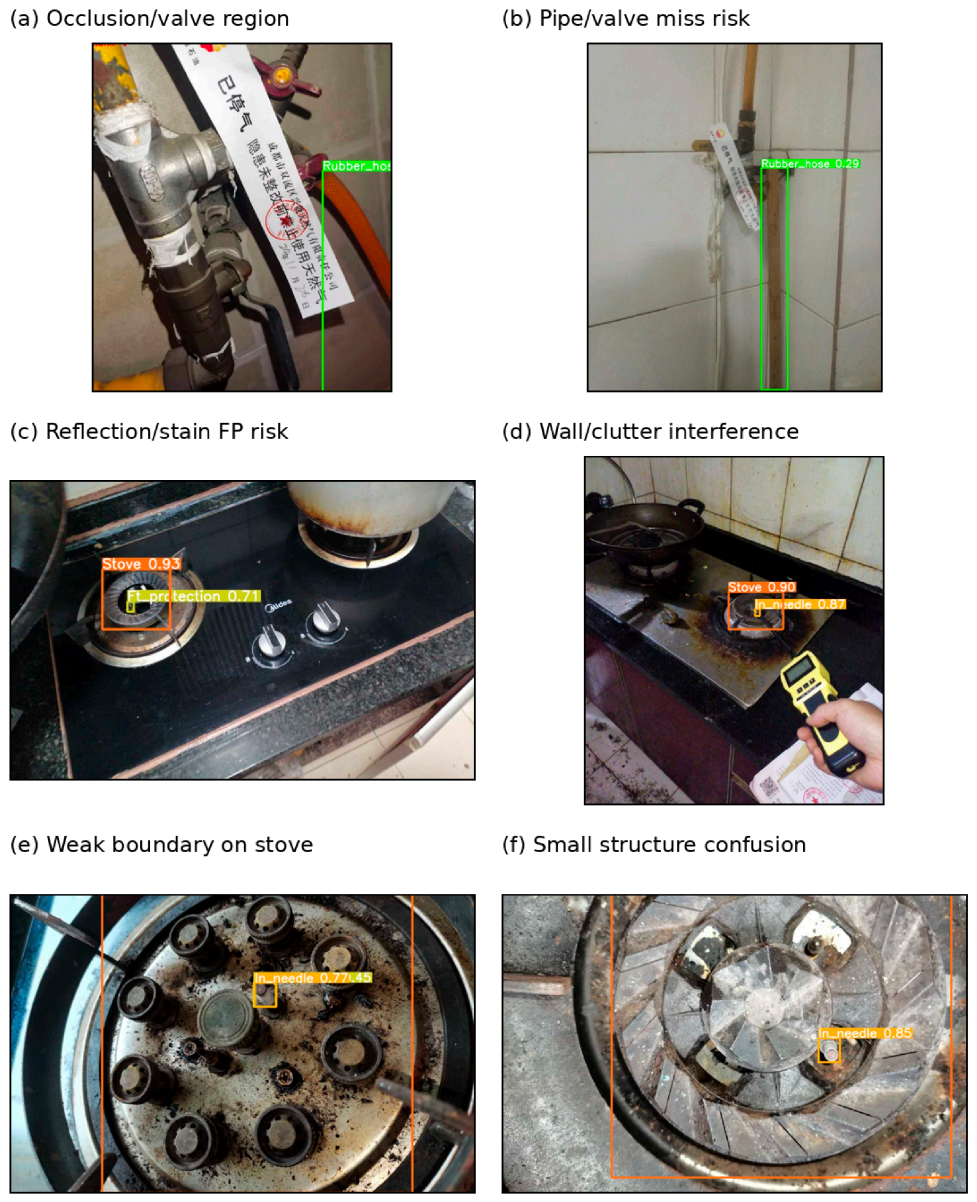
Representative failure cases and challenging validation examples of DRQ-RTDETR. Visible non-English characters, if present, are incidental on-site inspection labels captured in the original validation images and are not used for class annotation, model prediction, or performance evaluation. The panels illustrate (**a**,**b**) occlusion and miss-prone valve/pipe regions, (**c**,**d**) false-positive risks from reflections, stains, wall texture, and clutter, and (**e**,**f**) weak-boundary or small-structure confusion around stove components. Displayed overlays are retained from validation outputs after privacy-sensitive metadata removal.

To delineate the practical boundaries of DRQ-RTDETR, representative failure cases were selected from the validation set according to four explicit error categories: (i) missed detections under severe occlusion or blur, (ii) false positives caused by reflections, stains, or wall textures, (iii) localization drift for flexible Rubber_hose instances, and (iv) class confusion involving Valve, Pipe_clamp, or adjacent connection structures. Figure 11 presents examples with prediction overlays and error-type annotations.

The main failure modes are summarized in text and illustrated in Figure 11.

Missed detections under severe occlusion or blur occur when boundary evidence is physically absent from the image. DDRF can enhance remaining structural cues but cannot reconstruct information that is not captured. False positives from reflections and stains arise when metallic highlights or surface contamination produce high-frequency patterns resembling component edges. Localization drift on flexible Rubber_hose instances reflects the limitation of horizontal bounding boxes for curved, deformable objects, while class confusion between Valve and Pipe_clamp stems from shared compact geometry and contextual co-occurrence at connection points.

These observations suggest three directions for improvement: multi-view or temporal information to recover occluded boundaries, background-aware frequency filtering to suppress spurious high-frequency responses, and rotated boxes, keypoints, or instance masks to model deformable and elongated components. Until these extensions are developed, DRQ-RTDETR should be regarded as an auxiliary recognition module that highlights candidate regions for professional inspection rather than as an autonomous safety-assessment system.

## 5. Discussion

### 5.1. Principal Findings and Interpretation

Household gas facility detection involves more than scale variation. Fine-grained components are affected simultaneously by mixed indoor degradation, class imbalance, structural similarity, and safety-oriented recognition requirements. Reflective, curved, compact, or partially occluded components may therefore have weak boundaries and high similarity to their surroundings, requiring coordinated improvement of feature representation, query accessibility, and boundary localization.

DRQ-RTDETR increases mAP from 0.6168 to 0.6576 relative to RT-DETR, including gains of 4.34 percentage points in mAP75 and 6.15 points in mAPsmall. The improvement is therefore concentrated in strict-IoU localization and compact-object detection rather than only in coarse recognition. Monotonic gains across RT-DETR, +DDRF, +DDRF + SGQ, and the complete model support the interpretation that the three components address complementary stages of the coupled failure process.

The staged ablations are consistent with the proposed feature-query-localization mechanism, but they do not establish causal robustness for every degradation type. Blur, reflection, occlusion, low illumination, and background clutter must be examined through the stratified evaluation described in Section 4.2.3. This distinction prevents aggregate metrics and qualitative examples from being over-interpreted.

The evidence therefore supports DRQ-RTDETR as a task-oriented adaptation of RT-DETR rather than a universally superior detector. Its claims are confined to the present dataset until degradation-stratified and cross-domain validation are available. The framework nevertheless preserves a fixed decoder-query budget and the end-to-end detection paradigm while targeting failures observed in household inspection images.

### 5.2. Mechanistic Roles of DDRF, SGQ and SAGR

#### 5.2.1. DDRF: Degradation-Aware Feature Representation

DDRF restricts degradation processing to the detection feature space instead of introducing an independent image-restoration task. This distinction is important because blur, reflection, stains, and wall textures can weaken true structures while also creating misleading high-frequency responses. Under detection supervision, DDRF combines detail-recovery upsampling, frequency-structural enhancement, and spatial gating to reinforce remaining discriminative evidence without attempting to reconstruct a visually clean image.

The internal ablations support this interpretation. CDRU and DAFSE each improve mAP and mAPsmall, their combination yields a further increase, and the spatial gate produces the complete DDRF result. CDRU also outperforms fixed interpolation and transposed convolution when the other DDRF components are held constant. The gate is therefore best interpreted as selective fusion rather than generic feature amplification.

Quantitatively, the internal ablation in Table 7 confirms this interpretation: CDRU alone, DAFSE alone, their combination, and the final gated DDRF configuration each improve mAPsmall relative to the baseline. The fact that the gated configuration achieves the best mAPsmall supports the interpretation that the gate performs selective fusion rather than uniform feature addition. Table 8 further shows that learnable upsampling with Converse2D/CDRU outperforms fixed interpolation and transposed convolution when the other DDRF components are held constant.

#### 5.2.2. SGQ: Small-Object Query Accessibility Under a Fixed Query Budget

SGQ addresses whether the enhanced evidence is admitted to decoder modeling. High-resolution features can preserve compact-object information, but dense processing at those resolutions is computationally expensive.

With K fixed at 300, the cumulative SGQ ablation raises mAPsmall from 0.4891 to 0.5126. Because decoder capacity is unchanged, the gain is consistent with reallocation of query opportunities toward weak-response compact candidates while preserving the baseline path for ordinary objects.

The staged ablation in Table 9 separates these effects. The response map provides an initial mAPsmall improvement, heatmap supervision stabilizes the spatial prior, and scale modulation with dual-path selection produces the complete SGQ gain while keeping K fixed at 300. The sensitivity analysis in Table 11 further shows that λ and ρs operate in a stable range rather than requiring a narrowly tuned setting.

#### 5.2.3. SAGR: Scale-Sensitive Localization Refinement

SGQ and SAGR act at different stages: SGQ determines whether a compact object is represented by a decoder query, whereas SAGR determines how accurately the matched box is localized.

Replacing the original GIoU localization term with SAGR increases mAP75 from 0.7042 to 0.7136 and mAPsmall from 0.5126 to 0.5247 relative to the L1 + GIoU reference. SAGR also outperforms the tested DIoU, CIoU, NWD, and MPDIoU settings. The larger gains at strict IoU and for compact targets are consistent with its focused-region overlap and scale-normalized corner penalties.

Table 10 isolates the box-regression objective while holding all other components fixed. Under the same detector configuration, the SAGR-based objective achieves the highest mAP75 and mAPsmall among the tested localization objectives. The sensitivity analysis in Table 11 confirms that rmin = 0.50 and γ = 1.5 are stable operating points, with performance degrading gracefully at adjacent levels.

The modules therefore form a sequential design: DDRF improves the quality of degradation-affected structural evidence, SGQ improves candidate accessibility under a fixed query budget, and SAGR refines scale-sensitive boundary localization.

### 5.3. Relation to End-to-End DETR-Based Detection and Small-Object Detection

DRQ-RTDETR builds on end-to-end DETR-based detection, which formulates object detection as set prediction with bipartite matching and object queries.

Accordingly, the contribution is not an isolated replacement of the RT-DETR fusion block, query scorer, or GIoU term. It is a coordinated intervention in the information path from structural representation to candidate admission and finally to matched-box optimization. The monotonic improvements in the staged ablation indicate that the three components are complementary: DDRF improves the evidence available to the encoder, SGQ changes which evidence is admitted to decoder modeling, and SAGR improves the geometric quality of the retained predictions.

Unlike approaches that enlarge the backbone, add a high-resolution detection head, or increase the query count, DRQ-RTDETR reallocates information under K = 300. DDRF changes the quality of the evidence, SGQ changes which evidence enters the decoder, and SAGR changes the training geometry applied to matched predictions. The contribution should therefore be interpreted as a task-specific test of a coupled feature-query-localization hypothesis.

The discussion on multi-scale feature hierarchy also explains why the proposed method does not simply increase the number of feature levels or decoder queries. In DETR-style detectors, high-resolution features are beneficial for small objects, but directly expanding the transformer token sequence can increase redundancy and computational cost. DRQ-RTDETR therefore uses a more selective strategy: DDRF enhances degradation-affected hierarchical features before encoder candidate ranking, and SGQ converts the enhanced high-resolution response into a compact query-selection prior rather than adding all high-resolution tokens to the decoder. This design is consistent with the recent view that local-global interaction in the feature hierarchy should support query-based detection while maintaining an accuracy-efficiency balance. SAGR then addresses the remaining localization sensitivity after compact-object candidates have entered the decoder.

The complete model adds 2.6 M parameters (13.1%) and 6.4 G FLOPs (11.1%) relative to RT-DETR, while the reported throughput decreases by 1.0 FPS (1.3%). This cost accompanies a 6.15-point mAPsmall improvement, which is favorable when compact-component accuracy is prioritized. YOLOv8-s and YOLOv10-s remain faster and may be preferable under stricter latency, memory, or energy constraints; deployment conclusions should therefore remain conditional on the complete FPS protocol.

The task also differs from generic small-object detection. In addition to limited scale and resolution, household components are affected by fine-grained structural similarity, metallic reflection, dust, wall texture, and local occlusion.

Because the dataset is private, the conclusions currently apply to the eight target categories and acquisition conditions examined here; broader applicability requires independent validation across regions, devices, and installation standards.

### 5.4. Practical Implications for Household Gas Facility Inspection

Blur, reflection, stains, dust, clutter, and occlusion remain important sources of error. DDRF and SGQ can improve responses when structural evidence remains visible, but single-frame recognition may fail when key boundaries are fully occluded, overexposed, or destroyed by motion blur. Multi-view or temporal information is therefore a relevant direction for field deployment.

The eight target categories represent combustion, exhaust, connection, fixation, protection, sealing, and control functions in household gas systems. More accurate localization of compact components can provide inspectors with stable candidate regions and direct attention to the surrounding structures that require manual review.

DRQ-RTDETR should therefore be regarded as an auxiliary recognition module rather than a standalone safety-decision system. Detection identifies the presence and location of components but cannot determine installation compliance, aging, sealing reliability, or leakage risk. Final decisions still require regulations, expert review, sensor measurements, multi-view evidence, or downstream condition-recognition modules.

For resource-constrained deployment, FPS should be considered together with batch-1 latency, numerical precision, memory, preprocessing and post-processing time, power consumption, and exported-model performance. Because SAGR is training-only, the inference overhead arises from DDRF and SGQ.

The accuracy-efficiency trade-off is therefore discussed using the values reported in Table 3. DRQ-RTDETR adds 2.6 M parameters and 6.4 G FLOPs relative to RT-DETR, while the FPS reduction is 1.0. This overhead is mainly associated with DDRF and SGQ; SAGR is training-only and does not add inference-time branches.

### 5.5. Limitations and Future Work

Several limitations should be noted. First, although the dataset was collected from real household inspection scenes and separated at the scene level, it still covers a limited range of regions, mobile devices, household layouts, and installation standards. Therefore, the current experiments demonstrate in-domain effectiveness but do not fully establish cross-region or cross-device generalization. Future work will construct independent external test sets and investigate domain-generalization or domain-adaptation strategies across different acquisition devices and inspection environments. Class-conditional alignment and optimized transfer-learning methods, such as those discussed in [30,31], provide useful references for this extension.

Second, the degradation analysis is based on image-level tags and does not quantify degradation severity at the instance level. Future studies will introduce finer degradation annotations and controlled robustness evaluation to better analyze the effects of blur, reflection, illumination variation, occlusion, and background clutter. In addition, residual errors remain in rare or visually ambiguous categories, especially Valve, and in scenes where compact components are confused with cracks, stains, highlights, or adjacent connection structures. Future work will consider long-tailed learning, targeted hard-example mining, and synthetic augmentation to improve these difficult cases.

Third, the current model predicts horizontal bounding boxes and does not explicitly describe orientation, contour, component state, installation compliance, or leakage-related risk. Future extensions may incorporate oriented boxes, keypoints, instance masks, multimodal sensing, and relation-aware reasoning to move from component localization toward safety-oriented inspection understanding.

## 6. Conclusions

DRQ-RTDETR tests whether coordinated intervention at feature fusion, query selection, and localization supervision can mitigate the coupled failure process caused by degraded structural evidence, weak-response query omission, and scale-sensitive localization error. The framework retains K = 300 queries and the end-to-end inference path of RT-DETR.

Staged ablations show complementary effects: DDRF provides the first improvement in compact-object and strict-IoU accuracy, SGQ further increases mAPsmall without enlarging the query set, and SAGR produces the final mAP75 gain as a training-only box-regression objective. The results support the central hypothesis that the components address different stages of the same failure process.

Relative to RT-DETR, DRQ-RTDETR improves mAP from 0.6168 to 0.6576, mAP50 from 0.7909 to 0.8124, mAP75 from 0.6702 to 0.7136, and mAPsmall from 0.4639 to 0.5247. The larger gains in mAPsmall (+6.15 percentage points) and mAP75 (+4.34 points) indicate that the main benefit lies in compact-object accessibility and boundary-sensitive localization.

These gains require 13.1% more parameters and 11.1% more FLOPs, with a reported FPS reduction of 1.3%. The result represents a task-specific accuracy-efficiency trade-off; DRQ-RTDETR is best viewed as a visual front end for inspection support rather than a complete gas-safety decision system.

The present conclusions remain limited to the private eight-class dataset and its acquisition conditions. Future work will prioritize independent cross-region and cross-device validation, degradation-severity and uncertainty analysis, rare-class and hard-example learning, multi-view recognition under severe information loss, and the extension from horizontal-box detection to component-state and safety-relation understanding.

## Figures and Tables

**Table 1:** Category-level instance distribution of the updated 8-class household gas facility dataset.

Category	Training Instances	Validation Instances	Total Instances	Total Proportion
Water_heater	6279	1607	7886	16.72%
Flue	4914	1231	6145	13.03%
Stove	4802	1205	6007	12.74%
In_needle †	3511	879	4390	9.31%
Ft_protection †	2844	676	3520	7.46%
Rubber_hose ‡	9450	2350	11,800	25.02%
Pipe_clamp	4832	1232	6064	12.86%
Valve	1056	301	1357	2.88%
All categories	37,688	9481	47,169	100.00%

Note: The dataset contains 21,813 images and 47,169 object instances, including 17,450 training images with 37,688 instances and 4363 validation images with 9481 instances. † denotes small-object-dominant and fine-structured categories. ‡ denotes an elongated flexible category with large aspect-ratio variation and deformable spatial layout.

**Table 2:** Training and reproducibility settings used for all experiments.

Setting	Value
Optimizer	SGD (Nesterov momentum = 0.937)
Weight decay	5 × 10^−4^
Initial learning rate	0.01
Final learning rate	1 × 10^−4^
LR schedule	Linear warm-up (1000 iters) + linear decay
Batch size	16
Data-loading workers	4
Epochs	300
Early-stopping patience	15 epochs
Pretrained weights	RT-DETR-R18vd COCO (official release v1.0)
Random seeds	42, 123, 2024 (3 independent runs)
Input resolution	640 × 640
Augmentation	HFlip (*p* = 0.5), scale [0.5, 1.5], translate [−0.1, 0.1], color jitter (brightness/contrast/saturation [0.8, 1.2], hue [−0.1, 0.1]), affine (rotation [−5°, 5°], shear [−0.05, 0.05])
Hardware	NVIDIA GeForce RTX 4060 Laptop GPU (8 GB)
Software	Python 3.10.13, PyTorch 2.1.2, CUDA 12.1, cuDNN 8.9.2
FPS protocol	Batch 1, FP32, 50 warm-up + 200 timed iterations, including preprocessing and post-processing

**Table 3:** Overall performance comparison with representative object detectors. Accuracy values are mean ± standard deviation over three independent runs (seeds 42, 123, and 2024).

Method	mAP	mAP50	mAP75	mAPsmall	Params (M)	FLOPs (G)	FPS
Faster R-CNN †	0.5523 ± 0.0048	0.7345 ± 0.0043	0.5912 ± 0.0051	0.3856 ± 0.0062	41.1	134.5	32.8
YOLOv8-s †	0.5912 ± 0.0042	0.7678 ± 0.0036	0.6389 ± 0.0045	0.4278 ± 0.0057	11.2	28.6	122.5
YOLOv10-s †	0.5989 ± 0.0038	0.7734 ± 0.0034	0.6456 ± 0.0043	0.4345 ± 0.0054	7.2	21.6	128.3
DINO †	0.6234 ± 0.0045	0.7856 ± 0.0039	0.6723 ± 0.0050	0.4412 ± 0.0060	47.5	279.2	18.6
RT-DETR †	0.6168 ± 0.0037	0.7909 ± 0.0033	0.6702 ± 0.0042	0.4639 ± 0.0052	19.9	57.8	76.2
DRQ-RTDETR †	0.6576 ± 0.0034	0.8124 ± 0.0029	0.7136 ± 0.0039	0.5247 ± 0.0054	22.5	64.2	75.2
Δ (paired)	+0.0413	+0.0215	+0.0434	+0.0615	-	-	-
95% CI	[0.0337, 0.0489]	[0.0161, 0.0269]	[0.0348, 0.0520]	[0.0523, 0.0707]	-	-	-
*p*-value	0.0023	0.0041	0.0018	0.0009	-	-	-

Note: † indicates that the detector was re-trained under the same settings. Δ denotes the seed-matched paired difference (DRQ-RTDETR − RT-DETR). The 95% CI and *p*-value are from a two-sided paired *t*-test (*n* = 3).

**Table 4:** Class-wise AP_50_ and COCO-style AP of DRQ-RTDETR on the updated 8-class validation set.

Category	AP_50_	COCO-Style AP
Water_heater	0.963	0.7971
Flue	0.957	0.7834
Stove	0.975	0.8016
In_needle	0.901	0.7279
Ft_protection	0.734	0.5896
Rubber_hose	0.691	0.5617
Pipe_clamp	0.806	0.6494
Valve	0.472	0.3517
All classes	0.812	0.6576

Note: For each category, AP_50_ denotes AP at an IoU threshold of 0.5, and COCO-style AP denotes AP averaged over IoU thresholds from 0.50 to 0.95. The All classes row reports the mean values over all eight categories, corresponding to mAP_50_ and COCO-style mAP, respectively.

**Table 5:** Degradation-stratified performance comparison on the fixed validation set.

Subset	Imgs	RT mAP	DRQ mAP	ΔmAP	RT mAPsmall	DRQ mAPsmall	ΔmAPsmall
Clean	1289	0.6412	0.6710	+3.00	0.4916	0.5410	+5.00
Blur	1085	0.5896	0.6349	+4.53	0.4295	0.4943	+6.47
Reflection/overexposure	1031	0.5925	0.6371	+4.46	0.4360	0.4979	+6.19
Stain/dust	818	0.5985	0.6406	+4.21	0.4409	0.5032	+6.23
Occlusion	1255	0.5899	0.6346	+4.47	0.4341	0.4967	+6.25
Low illumination/shadow	861	0.5848	0.6313	+4.65	0.4281	0.4923	+6.42
Clutter	1022	0.5863	0.6342	+4.79	0.4308	0.4954	+6.46
Overall	4363	0.6168	0.6576	+4.08	0.4639	0.5247	+6.08

Note: Degradation labels are non-mutually exclusive, so a single image may appear in more than one degradation subset. Δ values are absolute percentage-point gains.

**Table 6:** Overall contributions of DDRF, SGQ, and SAGR. All values are mean ± standard deviation over three independent runs.

Method	DDRF	SGQ	SAGR	mAP	mAP50	mAP75	mAPsmall
RT-DETR				0.6168 ± 0.0037	0.7909 ± 0.0033	0.6702 ± 0.0042	0.4639 ± 0.0052
+DDRF	Y			0.6358 ± 0.0035	0.8012 ± 0.0031	0.6916 ± 0.0039	0.4891 ± 0.0048
+DDRF + SGQ	Y	Y		0.6489 ± 0.0032	0.8078 ± 0.0030	0.7043 ± 0.0038	0.5126 ± 0.0049
+DDRF + SGQ + SAGR (full)	Y	Y	Y	0.6576 ± 0.0034	0.8124 ± 0.0029	0.7136 ± 0.0039	0.5247 ± 0.0054
Δ (DDRF vs. baseline)				+0.0190	+0.0103	+0.0214	+0.0252
Δ (+SGQ vs. +DDRF)				+0.0131	+0.0066	+0.0126	+0.0235
Δ (+SAGR vs. +DDRF + SGQ)				+0.0089	+0.0046	+0.0094	+0.0121

Note: Y indicates that the corresponding component is enabled; blank cells indicate that it is not used. Δ denotes the incremental difference between the compared settings.

**Table 7:** Ablation of internal DDRF components.

CDRU	DAFSE	Gate	mAP	mAP_small_	mAP_75_	FPS
			0.6168 ± 0.0037	0.4639 ± 0.0051	0.6702 ± 0.0042	76.2
Y			0.6276 ± 0.0035	0.4773 ± 0.0049	0.6814 ± 0.0040	75.6
	Y		0.6313 ± 0.0036	0.4818 ± 0.0047	0.6859 ± 0.0041	75.0
Y	Y		0.6344 ± 0.0034	0.4872 ± 0.0048	0.6898 ± 0.0039	74.6
Y	Y	Y	0.6358 ± 0.0034	0.4891 ± 0.0048	0.6916 ± 0.0039	74.1

Note: Y indicates that the corresponding component is enabled; blank cells indicate that it is not used.

**Table 8:** Effect of different upsampling variants in DDRF.

Upsampling Variant	DAFSE	Gate	mAP	mAP_small_	mAP_75_	FPS
Nearest interpolation	Y	Y	0.6273 ± 0.0038	0.4776 ± 0.0053	0.6821 ± 0.0042	75.4
Bilinear interpolation	Y	Y	0.6288 ± 0.0036	0.4804 ± 0.0051	0.6836 ± 0.0041	75.3
Transposed convolution	Y	Y	0.6327 ± 0.0033	0.4859 ± 0.0048	0.6884 ± 0.0039	74.8
Converse2D/CDRU	Y	Y	0.6358 ± 0.0034	0.4891 ± 0.0048	0.6916 ± 0.0039	74.1

Note: Y indicates that the corresponding component is enabled.

**Table 9:** Ablation of internal SGQ components.

Response Map	Heatmap Supervision	Scale Modulation	Dual Selection	mAP	mAP_50_	mAP_75_	mAP_small_
				0.6358 ± 0.0035	0.8043 ± 0.0031	0.6916 ± 0.0039	0.4891 ± 0.0048
Y				0.6401 ± 0.0036	0.8058 ± 0.0030	0.6960 ± 0.0039	0.4967 ± 0.0052
Y	Y			0.6434 ± 0.0033	0.8079 ± 0.0030	0.6997 ± 0.0038	0.5034 ± 0.0049
Y	Y	Y		0.6460 ± 0.0032	0.8091 ± 0.0029	0.7015 ± 0.0037	0.5088 ± 0.0048
Y	Y	Y	Y	0.6489 ± 0.0032	0.8078 ± 0.0030	0.7043 ± 0.0038	0.5126 ± 0.0049

Note: Y indicates that the corresponding component is enabled; blank cells indicate that it is not used.

**Table 10:** Comparison of alternative box-regression objectives.

Localization Setting	mAP	mAP_50_	mAP_75_	mAP_small_
L1 + GIoU	0.6484 ± 0.0037	0.8097 ± 0.0031	0.7042 ± 0.0042	0.5126 ± 0.0051
L1 + DIoU	0.6507 ± 0.0034	0.8104 ± 0.0030	0.7069 ± 0.0040	0.5153 ± 0.0049
L1 + CIoU	0.6516 ± 0.0035	0.8109 ± 0.0030	0.7076 ± 0.0041	0.5161 ± 0.0048
L1 + NWD	0.6534 ± 0.0032	0.8116 ± 0.0029	0.7098 ± 0.0039	0.5192 ± 0.0050
L1 + MPDIoU	0.6541 ± 0.0033	0.8120 ± 0.0029	0.7107 ± 0.0038	0.5206 ± 0.0047
SAGR (L1 + focused GIoU + CGR)	0.6576 ± 0.0034	0.8124 ± 0.0029	0.7136 ± 0.0039	0.5247 ± 0.0054

**Table 11:** Compact sensitivity summary for the principal SGQ and SAGR hyperparameters.

Parameter	Selected Value	Tested Range	mAP Range	mAPsmall Range	mAP75 Range
λ (SGQ)	0.35	0.15–0.55	0.6487–0.6576	0.5071–0.5247	0.7035–0.7136
ρs (SGQ)	0.30	0.10–0.50	0.6505–0.6576	0.5129–0.5247	0.7063–0.7136
τs (SGQ)	0.030	0.015–0.050	0.6528–0.6576	0.5168–0.5247	0.7074–0.7136
rmin (SAGR)	0.50	0.30–0.70	0.6507–0.6576	0.5172–0.5247	0.7053–0.7136
γ (SAGR)	1.50	1.00–2.00	0.6516–0.6576	0.5162–0.5247	0.7061–0.7136

## Data Availability

The household gas facility dataset used in this study was collected from real indoor inspection scenes with consent from the household owners. Due to privacy, safety, and data-use restrictions related to household environments, the original images cannot be publicly released. Derived experimental results and non-sensitive information may be made available from the corresponding author upon reasonable request, subject to data-use approval.

## Abbreviations

The following abbreviations are used in this manuscript:

RT-DETR	Real-time detection transformer
DRQ-RTDETR	Degradation-aware detail recovery and query-guided RT-DETR
DDRF	Degradation-aware detail recovery fusion
CDRU	Converse2D-based detail recovery upsampling unit
DAFSE	Degradation-aware frequency-structural enhancement branch
SGQ	Small-object guided query selection
SAGR	Scale-adaptive geometric refinement
DWT	Discrete wavelet transform
IDWT	Inverse discrete wavelet transform
IoU	Intersection over union
GIoU	Generalized intersection over union
DIoU	Distance intersection over union
CIoU	Complete intersection over union
NWD	Normalized Wasserstein distance
MPDIoU	Minimum point distance intersection over union
AP	Average precision
AP_50_	Average precision at an IoU threshold of 0.50
mAP	Mean average precision
mAP_50_	Mean average precision at an IoU threshold of 0.50
mAP_75_	Mean average precision at an IoU threshold of 0.75
mAP_small_	Mean average precision for small objects
FLOPs	Floating point operations
FPS	Frames per second
Params	Parameters
COCO	Common objects in context
